# Perithyroidal Adipose Tissue Drives Thyroid Tumorigenesis through Adipokine Signaling and Immune Suppression

**DOI:** 10.34133/research.1360

**Published:** 2026-07-14

**Authors:** Qiaoyun Long, Zhaoxi Zhang, Bo Lin, Zhehan Yu, Mingxi Xu, Shi-ting Zhao, Jie Li, Ping Gu, Mengyuan Xie, Xuemei Zhao, Tuo Deng, Weiqin Yang, Alfred S. Cheng, Wah Yang, Weiming Lv, Yongqin Pan, Hannah Xiaoyan Hui

**Affiliations:** ^1^ School of Biomedical Sciences, Faculty of Medicine, The Chinese University of Hong Kong, Hong Kong, China.; ^2^ Department of Thyroid Surgery, The First Affiliated Hospital of Sun Yat-Sen University, Guangzhou, China.; ^3^ The First Affiliated Hospital of Jinan University, Guangzhou, China.; ^4^ Department of Thyroid and Breast Surgery, Guangzhou Women and Children’s Medical Center, Guangzhou Medical University, Guangzhou, China.; ^5^ Department of Endocrinology, Jinling Hospital, School of Medicine, Nanjing University, Nanjing, China.; ^6^ National Clinical Research Center for Metabolic Diseases, and Department of Metabolism and Endocrinology, The Second Xiangya Hospital of Central South University, Changsha, Hunan 410011, China.; ^7^ Kunming Institute of Zoology– The Chinese University of Hong Kong (KIZ-CUHK) Joint Laboratory of Bioresources and Molecular Research of Common Diseases, The Chinese University of Hong Kong, Hong Kong, China.

## Abstract

The perithyroidal adipose tissue (PAT), given its direct anatomical proximity to the thyroid gland, has long been postulated as a modulator of the thyroid tumor microenvironment. However, its cellular composition, functional heterogeneity, and specific roles in thyroid cancer progression remain unknown. To address these knowledge gap, we performed single-nucleus RNA sequencing of PAT from patients with papillary thyroid carcinoma (PTC) and multinodular goiter (MNG), combined with machine learning, proteomics, immunofluorescence, ex vivo assays, and human serum analysis. We constructed the first high-resolution atlas of human PAT, revealing an immune-rich niche and previously unrecognized adipocyte heterogeneity, including thermogenic subpopulations (BL-Ad1, BL-Ad2, OXPHOS-Ad) from distinct progenitors. Functionally, the PAT secretome from PTC substantially enhanced thyroid cancer cell proliferation compared to MNG. Integrated analyses identified a pathogenic adipokine triad characterized by loss of ADIPOQ and gain of NAMPT and IGF1. Restoring ADIPOQ signaling or inhibiting NAMPT/IGF1 suppressed tumor growth in vitro and in vivo. Additionally, we identified CCL14, down-regulated in PTC-derived OXPHOS-Ad, as a key immune regulator. Reduced CCL14–CCR1 signaling impaired CD80 expression in M1-like macrophages, disrupting CD80–CD28 costimulation and consequently diminishing T cell proliferation and recruitment. Consistently, circulating CCL14 levels were reduced in PTC patients. In conclusion, PAT acts as a dynamic endocrine and immunomodulatory component of the tumor microenvironment that promotes thyroid tumor growth through adipokine-mediated and immune-dependent mechanisms.

## Introduction

Thyroid cancer represents one of the most common endocrine malignancies worldwide, with a rapidly increasing incidence over recent decades with the incidence increased almost 3-fold in the last 3 decades and an annual growth rate of 13% that shows a remarkable female predominance [1,2]. Among various histological subtypes, papillary thyroid carcinoma (PTC) accounts for approximately 80% of all cases, making it the primary focus of both clinical management and research efforts [3]. While established risk factors including female sex [4], radiation exposure [5], iodine deficiency [6], a history of benign thyroid disease [7], obesity [8], and genetic predisposition [9–11] have been identified, the molecular mechanisms driving thyroid carcinogenesis, especially the remodeling of the tumor microenvironment, remain poorly understood.

Emerging research has highlighted the importance of tumor microenvironment in cancer progression [12]. Adipose tissue, once considered merely an energy storage depot, is now recognized as a dynamic endocrine organ that secretes various adipokines with pleiotropic effects on cancer biology [13,14], including thyroid cancers [15–17]. Serum leptin levels are considerably higher in cases with PTC than those with benign thyroid nodules (BTNs) and serve as a potential tumor marker for PTC [18], whereas increased leptin receptor expression in PTC is associated with a more aggressive PTC phenotype [19]. On the other hand, clinical observations consistently demonstrate reduced circulating level of the beneficial adipokine adiponectin in thyroid cancer patients compared to healthy controls [15,20], suggesting a tumor-suppressive role of this adipokine. However, most existing studies have relied on association investigations on adipokine levels with thyroid carcinoma, which fail to establish causal relationships between adipose tissue and thyroid carcinogenesis.

Adipose tissue exhibits substantial heterogeneity in function and cellular composition [21,22]. Based on functional characteristics, adipose tissues are classified into white adipose tissue (WAT) and brown adipose tissue (BAT) [21,23]. While WAT is primarily responsible for energy storage, BAT is specialized for thermogenesis and energy expenditure, mediated by uncoupling protein 1 (UCP1)-elicited nonshivering thermogenesis [24]. Notably, studies in the past years have also revealed the presence of noncanonical, UCP1-independent thermogenic mechanisms achieved by various futile cycles, or characterized by high expression of oxidative phosphorylation genes (OXPHOS-Ad) [25,26]. Anatomically, adipose depots distributed throughout the body demonstrate nuanced but distinctive biological functions and cellular architectures [27–29]. The perithyroidal adipose tissue (PAT), residing in direct anatomical proximity to the thyroid gland, represents a critically understudied component in thyroid pathophysiology [30]. Though once regarded merely as an anatomical structure providing mechanical support to the thyroid gland, PAT is now postulated to be a key modulator of the thyroid tumor microenvironment, given its unique anatomical location that enables direct crosstalk with the thyroid gland [31]. Current evidence confirms that invasion into perithyroidal fat constitutes minimal extrathyroidal extension (mETE), a well-established adverse prognostic factor associated with higher tumor stage and increased risk of locoregional recurrence [30,32,33]. Nevertheless, fundamental aspects of PAT biology remain elusive, including its baseline cellular composition, potential remodeling in thyroid cancer, and functional roles in thyroid cancer development and progression.

Recent advances in single-cell resolution transcriptomic technologies, particularly single-nucleus RNA sequencing (snRNA-seq), have revolutionized our ability to decode cellular complexity within tissues [31,34]. These powerful tools enable construction of high-resolution cellular atlases, revealing distinct cell populations within different adipose tissue depots, such as mature adipocytes, adipose stem and progenitor cells (ASPCs), and immune cells [34]. Furthermore, the identification of cellular subpopulations and cells in different functional states has advanced our understanding of adipose tissue diversity and dynamic remodeling under healthy and diseased conditions [26,35,36]. The application of these technologies to various adipose depots has provided unprecedented insights into their distinct cell type-specific gene expression patterns and functional specialization [28,29].

In this study, we applied snRNA-seq to PAT obtained from female patients with multinodular goiter (MNG) and PTC to construct the first high-resolution cellular atlas of human PAT, and identify specific cell population changes associated with thyroid malignancy. Furthermore, integrated machine learning and ligand–receptor interaction analysis identified key secreted factors associated with thyroid malignancy. Through integration with public single-cell datasets of human thyroid tumors, we depicted the first ligand–receptor-mediated interactions between PAT and the cells within the thyroid gland and tumor. Our comprehensive analysis reveals PAT as a dynamic endocrine and immunomodulatory organ that undergoes remarkable remodeling in PTC, contributing to tumor progression through both direct effects on cancer cells and modulation of the immune microenvironment.

## Results

### snRNA-seq reveals the cellular landscape of PAT and its immune-rich character

**Fig. 1.**
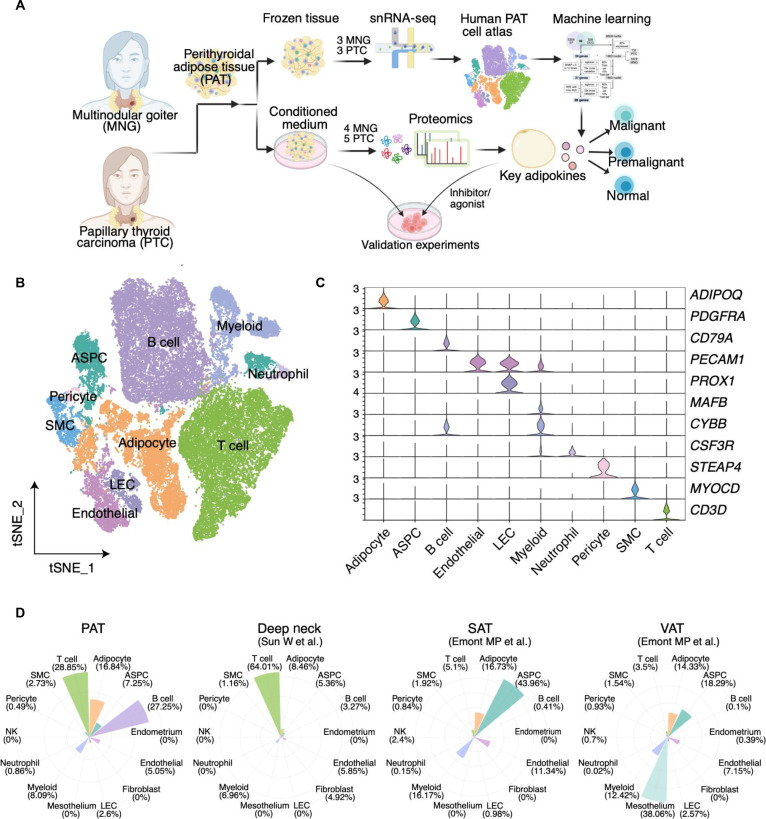
Establishment of a single-cell atlas of human perithyroidal adipose tissue (PAT). (A) Schematic of workflows for sample collection, processing, and bioinformatic analyses of human PATs from female patients with either multinodular goiter (MNG) or papillary thyroid carcinoma (PTC). (B) t-distributed stochastic neighbor embedding (t-SNE) plot of all 53,124 sequenced cells showing the cell types of human PAT. ASPC, adipose stem cell; SMC, smooth muscle cell; LEC, lymphatic endothelial cell. (C) Violin plot of the canonical marker genes for each cell population in the human PAT. (D) Relative proportion of each cell population in human PAT, deep neck fat, SAT, and VAT.

To gain a high-resolution understanding of the cellular landscape of PAT and its tumor-associated remodeling at the single-cell level, we conducted snRNA-seq on PAT specimens obtained from a cohort of female patients, comprising 3 with PTC and 3 with MNG as the benign control (Fig. 1A and Table S1). Following quality control, we obtained a final dataset of 53,214 high-quality nuclei, with a median detection of 1,640 genes per nucleus. Subsequent integration of data across all patients and clustering via t-distributed stochastic neighbor embedding (t-SNE) identified 10 principal cell populations (Fig. 1B). These populations were unequivocally annotated based on the expression of canonical marker genes, encompassing adipocytes, ASPCs, endothelial cells, T cell, B cell, myeloid cells, neutrophil, pericyte, lymphatic endothelial cell (LEC), and smooth muscle cell (SMC) (Fig. 1C).

We next sought to identify the distinctive features of PAT by comparing its cellular composition to other adipose depots. A comparative analysis was performed by integrating our snRNA-seq data with publicly available datasets on human deep neck fat [36], which is a major BAT depot in human [36,37], and human subcutaneous adipose tissue (SAT) and visceral adipose tissue (VAT) [34], representing the WAT in human. Interestingly, a shared immune-rich phenotype was revealed between PAT and deep neck fat, which is not prominent in human WATs (Fig. 1D). The deep neck depot was overwhelmingly composed of T cells (~64%), whereas PAT exhibited a balanced but substantial lymphoid compartment, with T cells and B cells accounting for 28.85% and 27.25% of all cells, respectively. Conversely, the adipocyte proportion in PAT aligned with that observed in SAT and VAT depots (Fig. 1D).

### PAT harbors heterogeneous adipocyte subpopulations with distinct functional identities

**Fig. 2.**
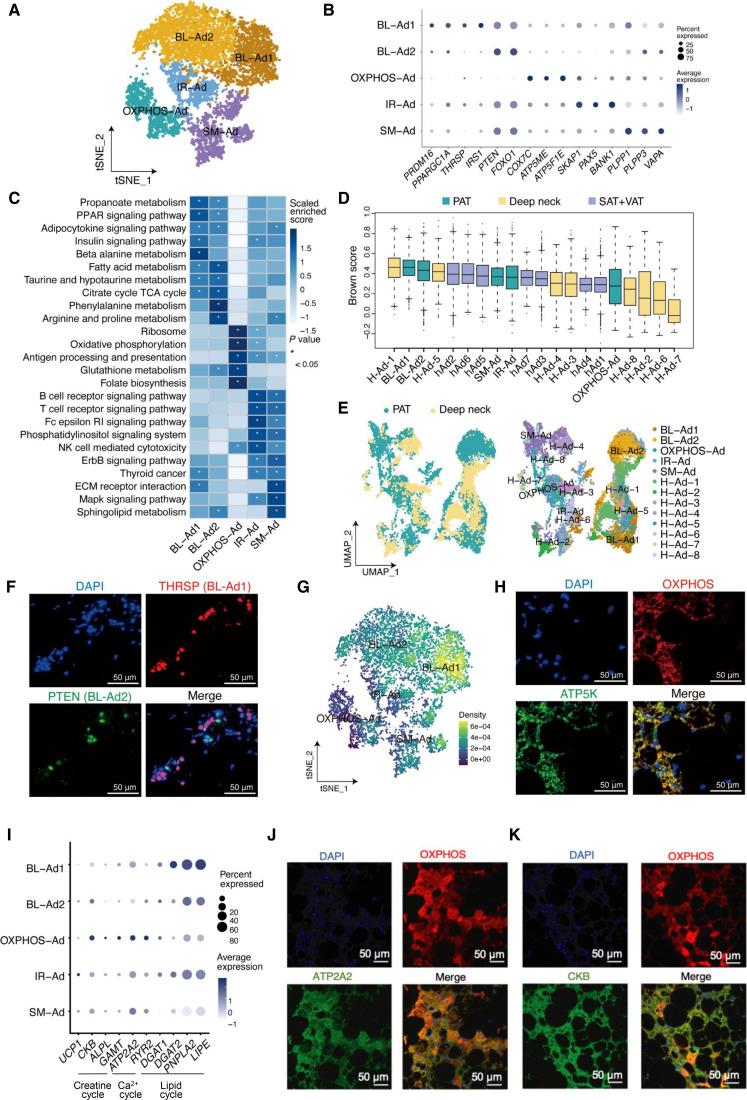
Adipocyte heterogeneity in human PAT. (A) t-SNE plot showing the adipocyte subclusters isolated from human PAT. (B) Dot plot illustrating the expression level of adipocyte marker genes in adipocyte subclusters. The size and color of the dots represent the proportion of expressing cells and the average expression level, respectively. (C) Results of gene set enrichment analysis (GSEA) in adipocyte subclusters. The plot displays the significantly enriched functional gene sets (with P < 0.05) for each subcluster. (D) Brown scores in adipocyte subpopulations in human PAT, deep neck fat, SAT, and VAT. This score assesses the thermogenic potential of adipocyte subpopulations. (E) UMAP of integrated adipocyte subpopulations in human PAT and deep neck fat, allowing for a direct comparison of their cellular composition and transcriptional similarity. (F) Representative immunofluorescence staining of *THRSP* and *PTEN* in human PAT (*n* = 3). (G) Thyroid hormone signaling score in adipocyte subclusters, derived from the expression of a panel of genes involved in this pathway. (H) Representative immunofluorescence staining of *ATP5K* and OXPHOS in human PAT (*n* = 3). (I) Dot plot of thermogenic gene expressions in adipocyte subclusters. (J and K) Representative immunofluorescence costaining of OXPHOS with ATP2A2 (J) and CKB (K) in human PAT (*n* = 3).

To further dissect the heterogeneity of adipocytes within PAT, we depicted a more detailed adipocyte map by subpopulation delineation. We categorized the adipocyte population into 5 distinct subpopulations that highly express classical adipocyte marker genes such as *PLIN4*, *PPARG*, and *ADIPOQ* (Fig. S1A). These subpopulations were then functionally annotated and named based on the expression profiles of their signature marker genes. Notably, one subpopulation exhibited prominent expression of immune response-related genes such as *SKAP1* and *BANK1*. Gene set enrichment analysis (GSEA) revealed that this subpopulation was primarily enriched in biological processes associated with the T cell receptor and B cell receptor signaling pathways (Fig. 2C). Given its unique immune-associated molecular features, this subpopulation was annotated as the “immune-responsive adipocyte” (IR-Ad) (Fig. 2B and C). Another adipocyte subpopulation was characterized by abundant expression of *PLPP1*, *PLPP3*, and *VAPA*, which are related to sphingolipid metabolic process (Fig. 2B and C). We therefore annotated this subpopulation as the “sphingolipid metabolic adipocyte” (SM-Ad), which is analogous to the subcluster hAd5 identified in human SAT and VAT [34].

Notably, we identified 2 distinct adipocyte subpopulations marked by an enrichment of *PRDM16*, a master regulator of brown adipocyte fate determination [38,39], as well as elevated expression of *PPARGC1A*, a central activator of mitochondrial biogenesis (Fig. 2B). Based on this transcriptional profile, we designated these clusters as the “brown-like adipocyte 1” (BL-Ad1) and “brown-like adipocyte 2” (BL-Ad2), respectively (Fig. 2B). To functionally benchmark their browning potential, we calculated their brown score based on a panel of human brown adipocyte marker genes [40]. Both BL-Ad1 and BL-Ad2 exhibited high brown scores, comparable to those of the established brown-like subpopulations H-Ad-1 and H-Ad-5 from human deep neck fat (Fig. 2D), confirming their thermogenic character. Cross-tissue integration of adipocyte subpopulations from PAT and deep neck fat further underscored the similarity between these populations. Dimensionality reduction revealed close spatial proximity between BL-Ad1/BL-Ad2 and H-Ad-1/H-Ad-5 (Fig. 2E), reflecting their high transcriptomic similarity. This finding was corroborated by cluster correlation analysis, which showed strong alignment among these 4 subpopulations (Fig. S1B).

Next, we validated the presence of BL-Ad1 and BL-Ad2 in PAT sections using their respective marker genes, *THRSP* and *PTEN*. Immunofluorescence staining showed a distinctive expression pattern of THRSP and PTEN in PAT (Fig. 2F). Furthermore, to explore the functional heterogeneity between the 2 subpopulations, we profiled their expression of thyroid hormone receptors *THRA* and *THRB*, which are known to modulate adipocyte browning [41,42]. BL-Ad1 coexpressed both receptors, whereas BL-Ad2 predominantly expressed *THRB* (Fig. S1C). Consistent with this pattern, BL-Ad1 showed higher expression of representative THRA/THRB target genes such as *CCND2* and *ACSL1*, while BL-Ad2 was enriched specifically in THRB target genes (Fig. S1C). Furthermore, gene set activity analysis indicated that the thyroid hormone receptor signaling was most active in BL-Ad1 (Fig. 2G), suggesting heightened sensitivity to thyroid hormone signaling in this subpopulation. Together with its higher brown score relative to BL-Ad2 (Fig. 2D), these data position BL-Ad1 as a more advanced brown-like adipocyte subpopulation within PAT.

Beyond these 2 brown-like adipocytes, we identified a distinct thermogenic subpopulation within PAT characterized by signature enrichment of oxidative phosphorylation (OXPHOS) pathway genes, including *UQCRB* and *ATP5F1E* (Fig. 2B and C). We accordingly designated this cluster OXPHOS-Ad. Its presence in PAT was confirmed via immunofluorescence staining for the OXPHOS complex and its marker gene *ATP5K* (Fig. 2H). This subpopulation is transcriptionally aligned with the H-Ad-3 cluster of human deep neck fat, which is reported to perform thermogenesis through UCP1-independent futile cycles and similarly exhibits high *ATP5K* and low *UCP1* and *CPEB2* expression (Fig. S1D and E) [26]. In line with this mechanistic parallel, OXPHOS-Ad also showed elevated expression of key genes governing both the creatine (*CKB*, *ALPL*, *GAMT*) and calcium (*ATP2A2*, *RYR2*) futile cycles (Fig. 2I). Furthermore, immunofluorescence costaining of key enzymes involved in UCP1-independent futile cycles showed that OXPHOS-positive adipocytes exhibited robust colocalization with CKB, a key enzyme mediating the creatine futile cycle, as well as ATP2A2, a central regulator of calcium cycling (Fig. 2J and K). Importantly, adipocytes with low OXPHOS signal displayed minimal expression of both CKB and ATP2A2, indicating that the enrichment of these thermogenic enzymes is tightly associated with the OXPHOS-Ad population (Fig. S1F and G). Together, these data provide protein-level evidence that OXPHOS-Ad adipocytes are selectively equipped with the core molecular machinery required for futile cycle-mediated thermogenesis. Combined with their transcriptional enrichment of OXPHOS and thermogenic gene programs, these findings substantiate the designation of OXPHOS-Ad as a UCP1-independent thermogenic adipocyte subpopulation within PAT.

### Trajectory analysis uncovers 2 distinct lineages for thermogenic adipocyte differentiation

**Fig. 3.**
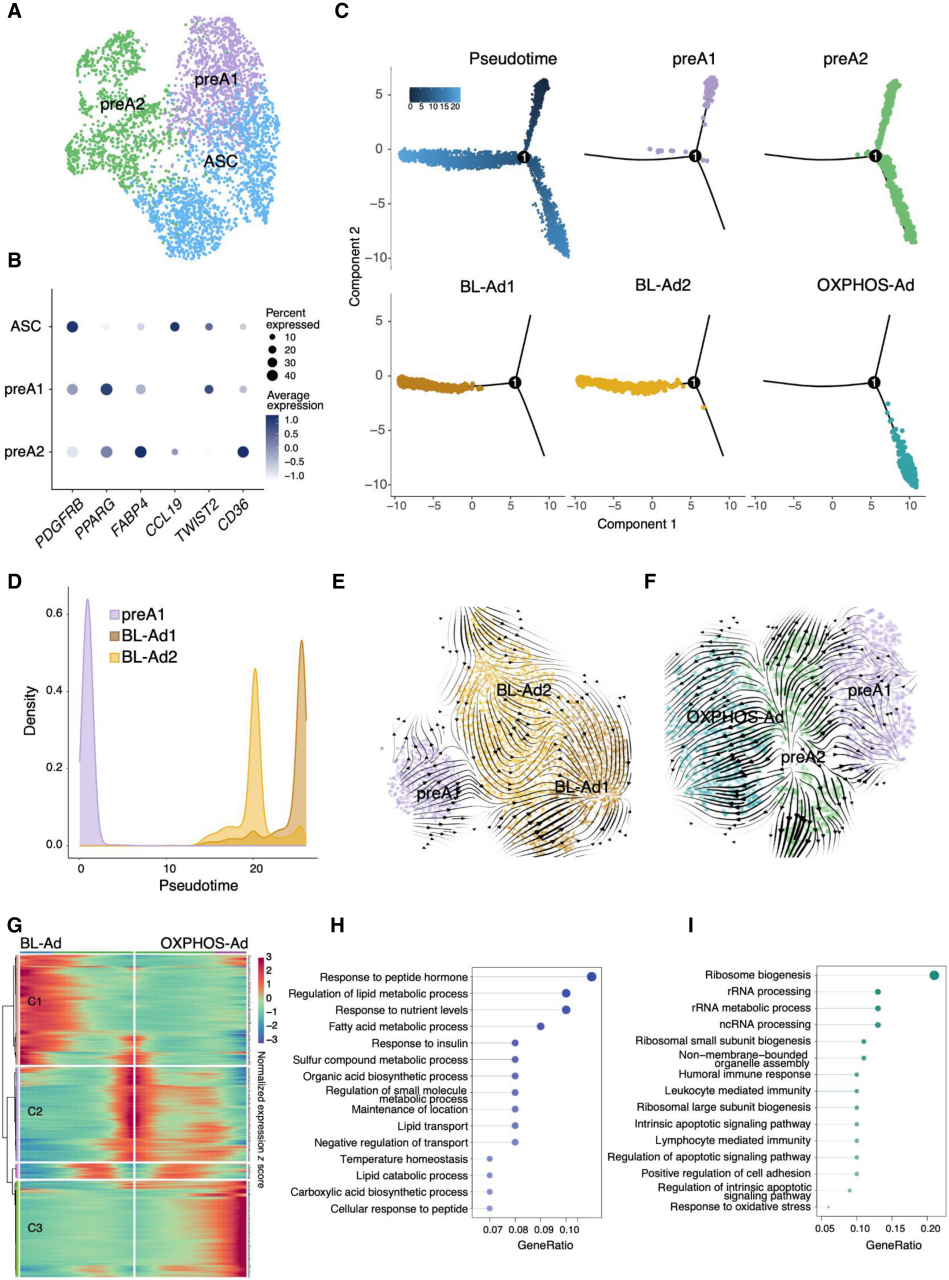
BL-Ad1/BL-Ad2 and OXPHOS-Ad separate into 2 trajectories. (A) UMAP plot displaying the subclusters of ASPCs isolated from human PAT. (B) Dot plot validating the identity of ASPC subpopulations by showing the expression of established marker genes for ASCs and preadipocytes (preA). (C) Trajectory among ASPC and adipocyte subpopulations, which reconstructs the potential developmental paths and branching points during adipocyte differentiation, suggesting distinct lineage relationships. (D) Cell density distribution along the inferred pseudotime trajectory for subclusters BL-Ad1, BL-Ad2, and preA1. The peaks indicate the relative positioning of these cell states along the differentiation continuum, with pseudotime representing a quantitative measure of cellular progression. (E and F) RNA velocity vector fields overlaid on the UMAP embedding. (E) Velocity streamlines for the BL-Ad1, BL-Ad2, and preA1 subclusters, predicting the future state and direction of cellular differentiation based on the ratio of unspliced to spliced mRNAs. (F) Similarly, velocity analysis for the OXPHOS-Ad, preA2, and preA1 subclusters, revealing a distinct differentiation flow separate from the BL-Ad lineage. (G) Heatmap of genes whose expression is dynamically changed along the pseudotime trajectory. These genes were clustered based on their expression patterns, revealing modules of genes associated with different stages of differentiation. (H and I) GO enrichment analysis of genes highly expressed in each of the 2 primary differentiation branches. (H) Biological processes significantly enriched in the gene set specific to the BL-Ad branch. (I) Biological processes significantly enriched in the gene set specific to the OXPHOS-Ad branch.

Given that BL-Ad1, BL-Ad2, and OXPHOS-Ad represent distinct thermogenic adipocyte types, we hypothesized that they originate from separate progenitor cells. To test this, we first subclustered the ASPC population, identifying 3 progenitor subsets, including 1 adipose stem cell (ASC) cluster and 2 preadipocyte clusters (preA1 and preA2) (Fig. 3A and B and Fig. S2A). Trajectory analysis revealed 2 principal differentiation pathways (Fig. 3C). The first branch delineated a lineage from preA1 to BL-Ad2 and terminally to BL-Ad1, with pseudotime ordering and RNA velocity both indicating that BL-Ad2 differentiates into BL-Ad1 (Fig. 3C to E and Fig. S2B). The second branch showed a commitment from preA2 to OXPHOS-Ad (Fig. 3C), a direction strongly supported by RNA velocity (Fig. 3F). We next identified genes dynamically expressed along pseudotime. Gene Ontology (GO) enrichment analysis showed that late-stage genes in the BL-Ad branch (cluster C1; Fig. 3G) were associated with fatty acid metabolism and insulin response (Fig. 3H), mirroring pathways enriched in mature BL-Ad1/2 (Fig. 2C). In contrast, genes up-regulated in the OXPHOS-Ad branch (cluster C3; Fig. 3G) were linked to ribosome biogenesis (Fig. 3I), consistent with the enriched pathways in this population (Fig. 2C). Collectively, these analysis revealed 2 distinctive differentiation pathways from different adipocyte precursors to the distinct thermogenic adipocyte subtypes BL-Ad1/BL-Ad2 and OXPHOS-Ad.

### PAT secretome promotes thyroid cancer cell growth in a paracrine manner

**Fig. 4.**
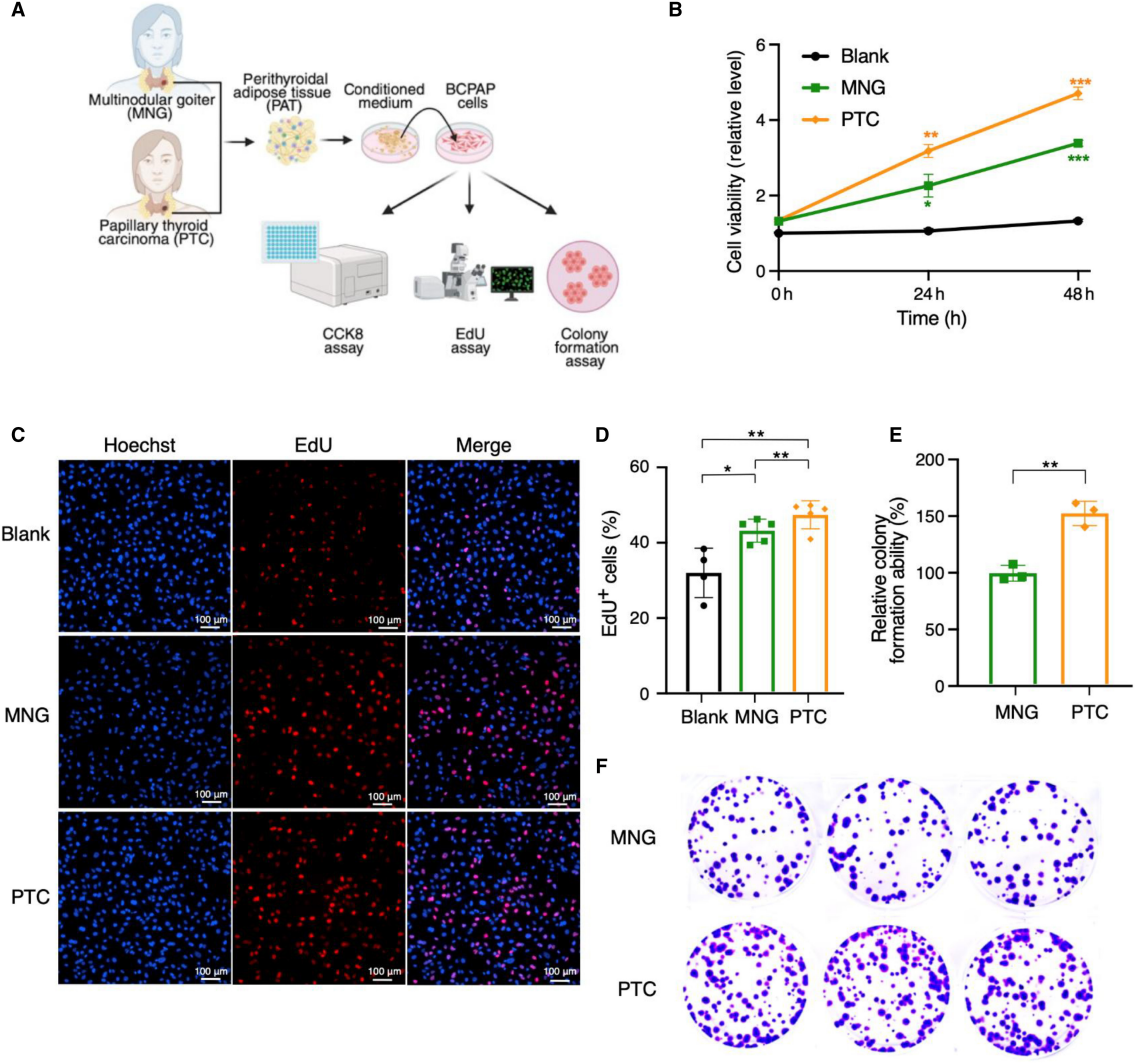
PAT from PTC patients enhances thyroid cancer cell proliferation via a paracrine manner. (A) Schematic of the experimental procedures. Human PAT was freshly isolated from patients. CM was collected and used to culture BCPAP cells for 24 h before analysis. (B) BCPAP cell viability measured by CCK-8 assay (*n* = 3). (C) Representative images of EdU incorporation in BCPAP cells. (D) Quantification of EdU-positive cells (n_blank = 4, n_MNG = 5, N_PTC = 5). (E) Plate colony formation assay to measure the colony formation ability of the BCPAP cells (*n* = 3). (F) Relative colony formation ability of the BCPAP cells (*n* = 3). Data are presented as means ± SEM. *P < 0.05, **P < 0.01.

To directly evaluate the functional role of PAT in the thyroid tumor microenvironment, we investigated whether PAT-secreted factors influence the growth of thyroid cancer cells. To this end, we established an ex vivo approach by collecting conditioned medium (CM) from the PAT of patients with either PTC or MNG, and treated the human PTC-derived cell line BCPAP (Fig. 4A). Cell proliferation was quantitatively assessed using multiple complementary assays. The Cell Counting Kit-8 (CCK-8) assay, which measures cellular metabolic activity as a proxy for viability, revealed a significant increase in absorbance at 450 nm in CM-treated groups compared to the control (Fig. 4B). Notably, PTC-CM induced a substantially greater proliferative response than MNG-CM at both 24- and 48-h time points (Fig. 4B). Furthermore, 5-ethynyl-2′-deoxyuridine (EdU) incorporation assay was performed to specifically quantify DNA replication in proliferating cells. Consistent with the CCK-8 results, the percentage of EdU-positive BCPAP cells was significantly higher in the CM-treated groups, with the PTC-CM group exhibiting the most potent effect (Fig. 4C and D). To assess long-term clonogenic survival and proliferative capacity, a colony formation assay was conducted. BCPAP cells were cultured for 24 d in the respective media, and the resulting colonies were stained and counted. This assay further corroborated the stronger pro-tumorigenic effect of the PAT from PTC patients, which revealed a significant increase in colony numbers upon treatment with PTC-CM (Fig. 4E and F). These findings indicate that PAT secretes transferable factors capable of stimulating thyroid cancer cell growth, and that this pro-proliferative activity is substantially enhanced in the context of PTC, suggesting a tumor-promoting shift in the PAT secretome.

### Machine learning identifies key adipocyte-derived secreted factors in PTC

**Fig. 5.**
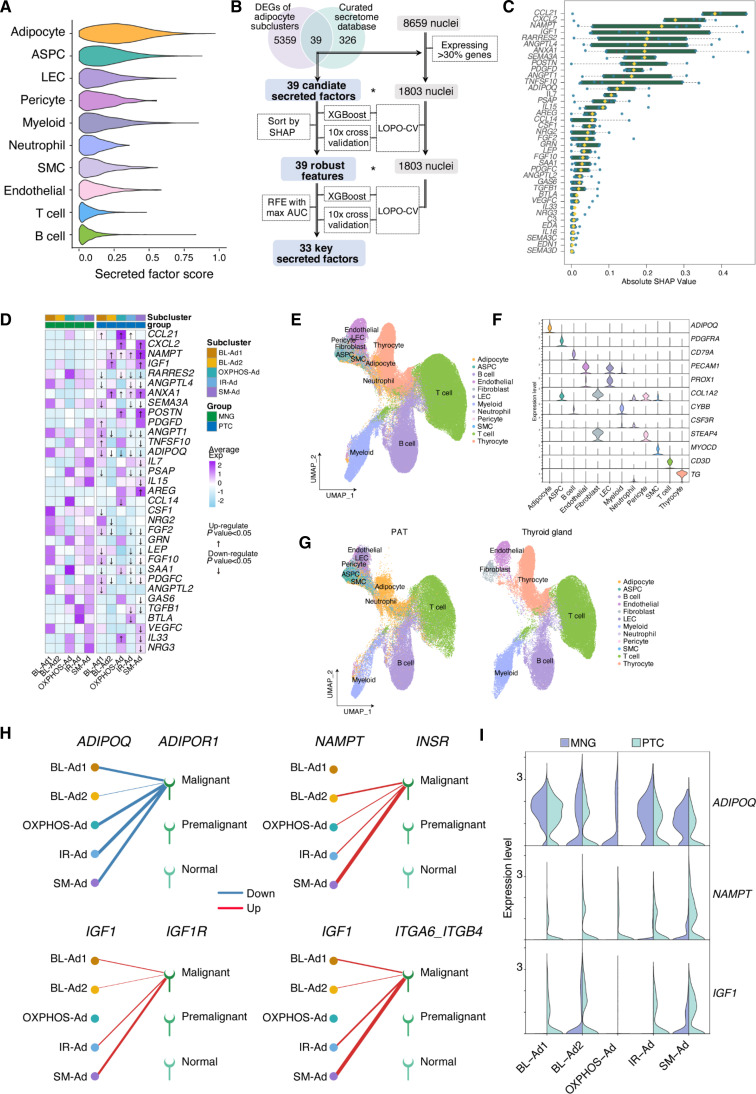
Potential secreted factors involved in the progression of PTC. (A) Violin plot showing the “Secreted Factor Score”, based on the expression of genes encoding secreted proteins across different cell populations. (B) Schematic diagram outlining the machine learning-based screening pipeline used to prioritize secreted factors linked to PTC progression. (C) Violin plots depicting the sorted SHAP values of 39 genes. Blue dots represent the SHAP values corresponding to LOPO samples, and yellow dots represent the mean SHAP values. (D) Heatmap visualizing the expression patterns of the differentially expressed secreted genes (DEGs) across the previously identified adipocyte subclusters. (E) UMAP plot displaying the main cell populations in thyroid tissue from the publicly available data downloaded from Pu et al. [43]. (F) Violin plot of the canonical marker genes for each cell population in the PAT and human thyroid tissue dataset. (G) UMAP plot displaying the main cell populations in thyroid split by tissue type. (H) Network diagram summarizing the predicted receptor–ligand interactions between the prioritized secreted factors from PAT adipocytes and their cognate receptors expressed on thyroid cancer cells. (I) Violin plots illustrating the expression distribution of *ADIPOQ*, *NAMPT*, and *IGF1* across adipocyte subclusters.

To systematically identify the PAT-derived molecular mediators responsible for its tumor-promoting effects, we first evaluated the secretory potential of each cell population within PAT. Analysis of secretory scores revealed that adipocytes exhibited the most pronounced secretory signature among all cellular constituents (Fig. 5A), designating them as the principal source of PAT-derived paracrine factors. We therefore focused subsequent analyses on the adipocyte compartment.

We employed an integrated machine learning strategy to prioritize functionally relevant secreted factors from adipocytes (Fig. 5B). Our approach began with intersecting 5,359 differentially expressed genes (DEGs) across adipocyte subpopulations [MNG versus PTC, |FC| > 1.5, false discovery rate (FDR) < 0.05] with a curated list of secreted proteins from CellChat 1.6.1 database, yielding 39 candidate secretory factors differentially expressed in PAT adipocytes. We selected the adipocyte nuclei expressing at least 30% of these candidate genes and 1,803 nuclei constituting 731 and 1,072 nuclei from PTC and MNG patients, respectively. Using this dataset, we constructed a classifier based on the XGBoost algorithm to discriminate adipocytes derived from PTC versus MNG patients.

To minimize the influence of interindividual variability, we implemented a leave-one-patient-out cross-validation (LOPO-CV) strategy throughout model training, feature selection, and evaluation (Fig. 5B). Across all LOPO-CV iterations, the model consistently achieved robust performance, with a mean area under the curve (AUC) exceeding 0.8 (Fig. S3A), indicating robust discriminatory performance. In parallel, 10-fold cross-validation was applied to further improve model stability and mitigate overfitting (Fig. 5B). To identify key predictive features, SHAP (SHapley Additive exPlanations) values were computed for each trained model within the LOPO-CV framework and subsequently averaged across iterations to derive robust and unbiased feature importance scores. Based on mean SHAP values, the 39 candidate secretory factors were ranked according to their contribution to classification performance (Fig. 5C).

We next applied recursive feature elimination (RFE), a widely used feature selection method [43], incorporating LOPO-CV in each iteration to ensure robustness against patient-specific effects. This analysis identified an optimal gene set comprising 33 genes, which achieved the highest predictive performance with an AUC of 0.912 (Fig. S3B). To further assess model robustness, we performed permutation testing, which confirmed that the predictive performance of the 33-gene signature was significantly higher than that obtained from randomly permuted class labels (Fig. S3C), indicating that the model is not driven by chance or sample-specific bias.

Interestingly, further analysis of the expression patterns of these 33 candidate genes across adipocyte subpopulations revealed that most genes exhibited distinct subpopulation-specific enrichment (Fig. 5D). For example, *ANGPTL4*, *SEMA3A*, *ANGPTL1*, *ADIPOQ*, *NRG2*, *CSF1*, and *LEP* were predominantly expressed and down-regulated in BL-Ad1 cells, whereas *CCL21*, *RARRES2*, *CCL14*, and *SAA1* were primarily enriched and differentially expressed in OXPHOS-Ad cells (Fig. 5D).

To characterize the crosstalk between PAT and the thyroid tumor microenvironment, we integrated our PAT snRNA-seq data with a published single-cell dataset of human thyroid tumors and para-tumor thyroid tissues (GSE184362) [43] (Fig. 5E to G). Within this integrated dataset, thyrocytes were resolved into normal, premalignant, and malignant subpopulations (Fig. S4A). The identity of malignant thyrocytes was characterized by a reduced thyroid differentiation score (TDS), down-regulation of thyroid-specific genes (*TPO*, *TG*, and *IYD*), and elevated *TMSB4X* expression (Fig. S4B and C), consistent with the original publication [43].

We next sought to identify direct paracrine signaling from PAT adipocytes to thyrocytes. To this end, we systematically performed ligand–receptor interaction analysis between all candidate secreted factors identified from adipocyte subpopulations and thyrocyte populations. Although multiple ligands exhibited differential expression across adipocyte subtypes (Fig. 5D), only *ADIPOQ*, *NAMPT*, and *IGF1* demonstrated the capacity to engage in direct tumor–adipocyte communication and formed ligand–receptor pairs with cognate receptors that are robustly expressed on malignant thyrocytes (Fig. 5H). In contrast, the rest of the candidate ligands displayed limited or negligible interaction potential due to low or absent expression of their corresponding receptors in thyrocytes. These results indicate that, among the differentially expressed adipocyte-derived factors, *ADIPOQ*, *NAMPT*, and *IGF1* represent the principal mediators of direct paracrine crosstalk between PAT and thyroid cancer cells within the local tumor microenvironment.5

Consistent with its established role as a tumor-suppressive adipokine, *ADIPOQ* was broadly expressed across all PAT adipocyte subpopulations, with the highest levels detected in BL-Ad1 (Fig. 5D). In PTC patients, however, *ADIPOQ* expression was significantly down-regulated compared to MNG controls (Fig. 5D and I). This reduction corresponded to a weakened ADIPOQ-ADIPOR1 interaction between PAT adipocytes and thyroid cells (Fig. 5H). In contrast, the expression of *NAMPT* (encoding visfatin), an adipokine known to promote cancer proliferation via insulin receptor (INSR) phosphorylation and subsequent mitogen-activated protein kinase (MAPK)/phosphatidylinositol 3-kinase (PI3K)–AKT pathway activation [44], was elevated in several adipocyte subpopulations especially in SM-Ad in PTC (Fig. 5D), leading to a stronger predicted NAMPT-INSR interaction in the tumor microenvironment (Fig. 5H and I). Similarly, IGF1, a recognized mitogenic factor [45], showed marked up-regulation in BL-Ad2 and SM-Ad under PTC condition (Fig. 5D and I). This increase was associated with enhanced communication via IGF1–IGF1R and IGF1–ITGA6_ITGA4 interactions in tumor samples (Fig. 5H). Taken together, these results indicate that PAT in PTC undergoes a pathogenic rewiring of its adipokine profile, characterized by the loss of the tumor-suppressive signal *ADIPOQ* and the simultaneous gain of the oncogenic factors *NAMPT* and *IGF1*, which likely coordinate to drive thyroid cancer cell proliferation.

### PAT-derived ADIPOQ, NAMPT, and IGF1 modulate thyroid cancer progression

**Fig. 6.**
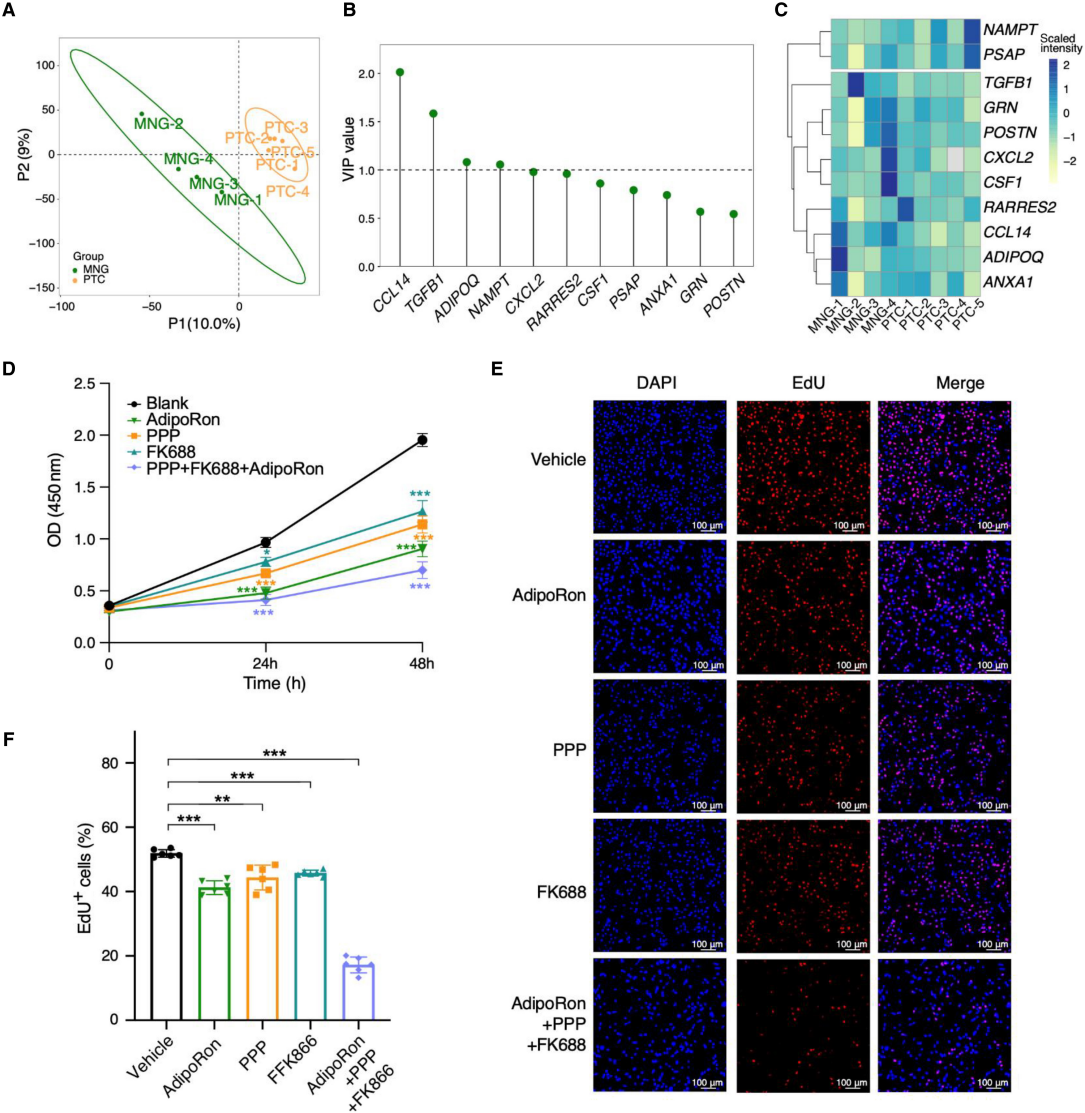
PAT-derived proteins modulate the proliferation of thyroid cancer cells. (A to C) PAT CM from NMG and PTC patients were subjected to proteomics analysis (n_MNG = 4, n_PTC = 5). (A) Partial least squares-discriminant analysis (PLS-DA) of the proteomic profile of CM collected from PAT. The plot demonstrates a clear separation in the secretory protein profile between PAT from patients with NMG and PTC. (B) The scatter plot shows the variable importance in projection (VIP) scores of 11 key proteins from the PLS-DA model. These 11 proteins were all derived from the 33 candidate genes identified by the previous machine learning screening (Fig. 5), and this result confirms the importance of these genes at the protein level. (C) Heatmap displaying the relative abundance of the 11 validated secreted proteins in PAT CM from NMG and PTC patients. (D to F) BCPAP cells were cultured in human PAT CM supplemented with PPP (an IGF1R inhibitor targeting the IGF1 pathway), FK688 (a NAMPT inhibitor), and AdipoRon (an adiponectin receptor agonist). (D) Cell viability measured by CCK-8 assay (*n* = 3). (E) Representative images of EdU incorporation in BCPAP cells. (F) Quantification of EdU-positive cells (*n* = 6). Data are presented as means ± SEM. *P < 0.05, **P < 0.01, ***P < 0.001.

To validate the protein-level secretion of the candidate factors ADIPOQ, NAMPT, and IGF1, we performed proteomic analysis on CM derived from PATs of 5 PTC and 4 MNG patients. Partial least squares-discriminant analysis (PLS-DA) revealed a clear separation between PTC and MNG proteomic profiles, indicating fundamental differences in the PAT secretome between malignant and benign conditions (Fig. 6A). We next evaluated whether the 33 candidate genes identified through machine learning contributed to this separation at the protein level. Variable importance in projection (VIP) analysis of detected proteins encoded by these genes identified ADIPOQ and NAMPT as key discriminators (VIP > 1) between PTC and MNG groups (Fig. 6B). Consistent with the transcriptomic findings, ADIPOQ protein levels were decreased, while NAMPT levels were increased, in PTC-derived CM compared to MNG controls (Fig. 6C). IGF1 was not detected in the proteomic analysis, potentially due to low secretion levels or technical limitations.

To functionally validate the roles of ADIPOQ, NAMPT, and IGF1 in PAT-mediated tumor promotion, we investigated whether pharmacological modulation of these signaling axes counteract the proliferative effects of PTC-derived PAT CM on BCPAP cells. We employed AdipoRon (an AdipoR agonist), FK866 (a NAMPT inhibitor), and PPP (an IGF1R inhibitor) at their respective median inhibitory concentration (IC₅₀) concentrations (7.7, 2, and 2 μM; Fig. S5). Individual treatment with each modulator significantly suppressed BCPAP cell proliferation in a time-dependent manner, effectively reversing the growth-promoting activity of PTC-CM (Fig. 6D). Notably, the triple combination of these pharmacological reagents yielded the most potent inhibitory effect (Fig. 6D), suggesting their nonredundant functions in tumor cell proliferation. This finding was corroborated by EdU incorporation assays, which revealed that each single reagent treatment significantly reduced PAT CM-induced DNA synthesis, while the triple-reagent combination resulted in massive suppression of proliferative activity (Fig. 6E and F).

**Fig. 7.**
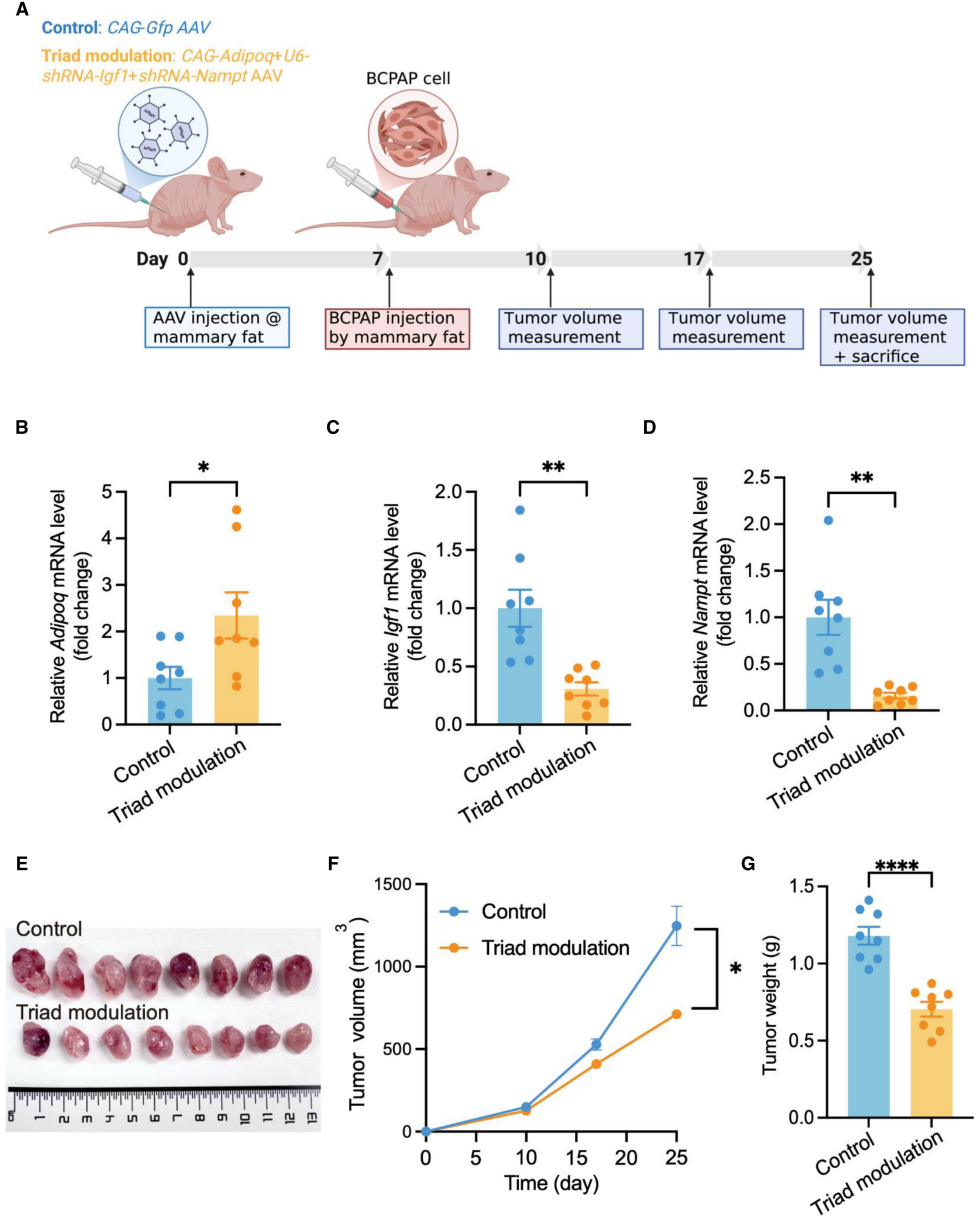
Targeted modulation of the adipokine triad suppresses PTC tumor growth in vivo. (A) Schematic diagram of the experimental workflow. Four-week-old female nude mice were randomly assigned to receive local AAV injection into the inguinal mammary fat pad. Control AAV (expressing GFP) or AAV encoding *Adipoq* together with shRNAs targeting *Igf1* and *Nampt* (triad modulation) was used. Seven days later, BCPAP cells were subcutaneously implanted adjacent to the AAV-targeted fat pad to establish xenograft tumors. (B to D) Relative mRNA expression levels of *Adipoq*, *Igf1*, and *Nampt* in peritumoral adipose tissue following treatment (*n* = 8). (E) Representative images of tumors from control and triad modulation groups (*n* = 8). (F) Tumor growth curves over time. (G) Tumor weights measured at day 25 (*n* = 8). Data are presented as means ± SEM. *P < 0.05, **P < 0.01, ****P < 0.0001.

To determine whether modulation of PAT-derived adipokines directly influences tumor growth in vivo, we employed an adeno-associated virus (AAV)-based strategy to alter adipokine expression in peritumoral adipose tissue (Fig. 7A). To recapitulate the adipokine profile observed in benign MNG, the fat pad adjacent to the transplanted tumors was locally injected with AAV that simultaneously overexpressed *Adipoq* and knockdown of *Igf1* and *Nampt*. Efficient modulation of the adipokine triad was confirmed by the real-time polymerase chain reaction (PCR) analysis of these genes in the targeted adipose tissues (Fig. 7B to D). Seven days after intra-adipose AAV injection, human PTC cells (BCPAP), a well-characterized thyroid cancer cell line, was injected to establish a xenograft model to ensure that modulation of *Adipoq*, *Igf1*, and *Nampt* occurred specifically within the adipose compartment, rather than in tumor tissue (Fig. 7A). Importantly, AAV-mediated modulation of the adipokine triad in the adipose tissue markedly attenuated tumor growth, as evidenced by reduced tumor volume and weight compared to control groups (Fig. 7E to G). Collectively, our integrated proteomic and functional analyses confirm that PAT in PTC exhibits a tumor-promoting secretory shift characterized by reduced ADIPOQ and elevated NAMPT expression. Furthermore, we demonstrate that either restoration of ADIPOQ signaling or inhibition of NAMPT and IGF1 pathways effectively suppresses thyroid cancer cell proliferation, with combined targeting yielding superior antitumor effects.

### CCL14 modulates CD80 expression in M1-like macrophages via the CCR1–PI3K–AKT–FOXO3 axis

**Fig. 8.**
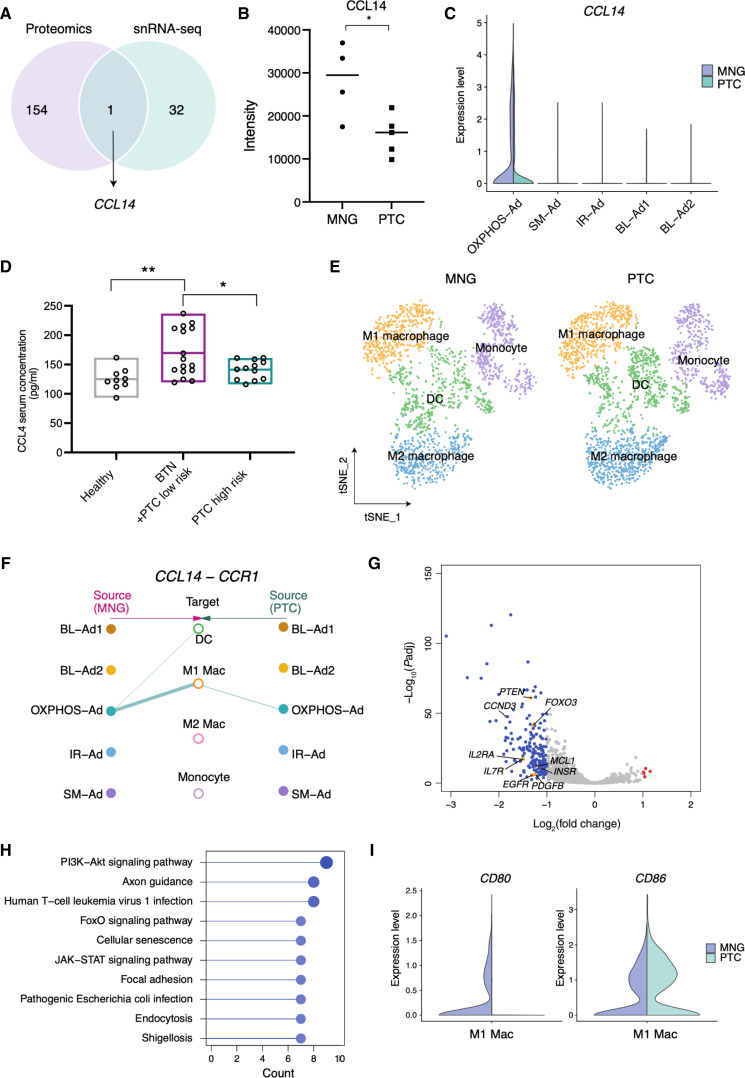
CCL14 is down-regulated in PAT from PTC patients. (A) Venn diagram illustrating the overlap between differentially expressed proteins identified in PAT CM and the 33 candidate genes identified by the machine learning screening, which identified CCL14 as a key molecule of interest. (B) Relative abundance of CCL14 in human PAT CM measured by proteomics (n_MNG = 4, n_PTC = 5). Mean values were shown. (C) Violin plot of *CCL14* gene expression levels from snRNA-seq data, comparing the expression levels among adipocyte subpopulations in human PAT samples from patients with MNG and those with PTC. (D) Serum concentration of CCL14 protein, as measured by ELISA in healthy individuals, patients with MNG, and patients with PTC (n_healthy = 9, n_BTN + low risk PTC = 16, n_high risk PTC = 12). Data are presented as floating bars showing the mean and the range (minimum to maximum). (E) UMAP plot of myeloid cell subclusters, colored by cell type and split by patient diagnosis (MNG versus PTC). (F) Predicted interaction strength of the CCL14–CCR1 ligand–receptor pair between adipocytes (source of CCL14) and myeloid cell subclusters (expressing the CCR1 receptor) in PAT from MNG versus PTC patients. (G) Volcano plot of DEGs in the M1-like macrophage subcluster when comparing PAT from PTC patients to MNG controls. (H) GO pathway enrichment analysis performed on the down-regulated DEGs from the M1-like macrophages. (I) Violin plots showing the expression distribution of the classic M1 macrophage markers *CD80* and *CD86* within the M1 macrophage subcluster. *P < 0.05, **P < 0.01.

To more systematically identify PAT-secreted protein factors identified by our proteomics analysis with potential roles in thyroid carcinogenesis, we integrated our proteomic data with 33 machine learning-prioritized secreted factors. This analysis identified C–C motif chemokine ligand 14 (CCL14) as the sole overlapping candidate (Fig. 8A and B). Notably, snRNA-seq data revealed that *CCL14* was specifically and highly expressed in the OXPHOS-Ad subpopulation, with significant down-regulation in PTC patients compared to MNG controls (Figs. 5D and 8C). This expression pattern was unique to PAT, as *CCL14* showed minimal expression in adipocyte subpopulations from deep neck fat, SAT, or VAT (Fig. S6A). Consistent with these transcriptomic findings, proteomic analysis of PAT-CM confirmed significantly reduced levels of CCL14 in PTC patients (Fig. 8C and Fig. S6B). Interestingly, thermogenesis pathway was significantly down-regulated in OXPHOS-Ad from PTC patients compared to those from MNG patients (Fig. S6C).

We further validated the clinical relevance of CCL14 reduction by measuring its serum levels across patient cohorts. Based on the 2015 American Thyroid Association (ATA) Guidelines and supported by established clinical practice, we categorized benign thyroid nodules (BTNs) and low-risk PTC together as a benign/indolent group, while high-risk PTC constituted the malignant group. Enzyme-linked immunosorbent assay (ELISA) analysis demonstrated that CCL14 was significantly elevated in the benign/indolent group compared to the healthy individuals (Fig. 8D). However, its serum concentration was significantly lower in high-risk PTC patients compared to the benign/indolent group (Fig. 8D), confirming the systemic down-regulation of CCL14 in aggressive thyroid cancer.

Given that CCL14 functions as a CC-type chemokine binding to CCR1, CCR3, and CCR5 receptors [46], we first examined these receptor expression patterns within thyroid tissue and PAT. We found predominant expression of *CCR1* among these receptors (Fig. S7A and B). Furthermore *CCR1* was abundantly expressed in myeloid cells within both thyroid tissue and PAT (Fig. S7A and B), suggesting potential CCL14-mediated immune regulation in myeloid cells. To investigate this, we subclustered PAT myeloid cells into 4 populations, including M1-like macrophages, M2-like macrophages, monocytes, and dendritic cells, with M1-like macrophage exhibiting a predominant *CCR1* expression (Fig. 8E and Fig. S7C and D). Although the MNG and PTC PAT had comparable number of M1-like macrophages (Fig. 8E), cell–cell interaction analysis identified a dramatic difference of CCL14–CCR1 interaction between these 2 conditions. While a robust direct CCL14–CCR1 interacting axis between OXPHOS-Ad and M1-like macrophages was detected in MNG patients, this interaction was attenuated in PTC patients (Fig. 8F). Similarly, intercellular communication analysis of PAT adipocytes with myeloid cells in thyroid tissues demonstrated a remarkably reduced CCL14–CCR1 axis under PTC condition (Fig. S7E and F).

To elucidate the functional role of the attenuated CCL14–CCR1 axis in PTC, we performed differential gene expression analysis on PAT-derived M1-like macrophages. Compared to MNG controls, M1-like macrophages from PTC patients exhibited a profound transcriptional down-regulation, with a number of genes showing reduced expression (Fig. 8G). Pathway enrichment analysis revealed that these down-regulated genes were prominently enriched in the PI3K–AKT signaling pathway (Fig. 8H). Key negative regulators of this pathway, including *PTEN* and the transcription factor *FOXO3*, were among the most significantly down-regulated genes (Fig. 7G), suggesting a potential dysregulation of this signaling cascade.

Given that CCR1 activation is known to trigger the PI3K–AKT pathway [47], the observed down-regulation of its ligand, CCL14, in PTC provides a plausible mechanism for the observed gene signature. We therefore hypothesized that the loss of CCL14-mediated signaling through CCR1 impairs PI3K–AKT activation, leading to downstream functional consequences. A critical downstream effector of PI3K–AKT signaling is FOXO3, a transcription factor whose activity is suppressed upon AKT-mediated phosphorylation. Notably, FOXO3 has been previously established as a direct regulator of *CD80* expression in macrophages [48]. In line with the down-regulation of *FOXO3*, we found that the expression of *CD80* was significantly reduced in M1-like macrophages from PTC patients (Fig. 8I). In contrast, the expression of CD86, a general M1 marker, remained unchanged, indicating a specific impairment of the CD80 costimulatory molecule rather than a broad loss of M1 identity. Collectively, these results delineate a specific signaling axis wherein the loss of CCL14 from OXPHOS-Ad in PTC disrupts CCR1-mediated PI3K–AKT signaling in neighboring M1-like macrophages. This disruption leads to decreased suppression of FOXO3, ultimately resulting in the specific down-regulation of CD80. This impaired CD80 expression may have important consequences for T cell costimulation and antitumor immunity.

### CCL14 deficiency impairs T cell activation via attenuated CD80–CD28 costimulation

**Fig. 9.**
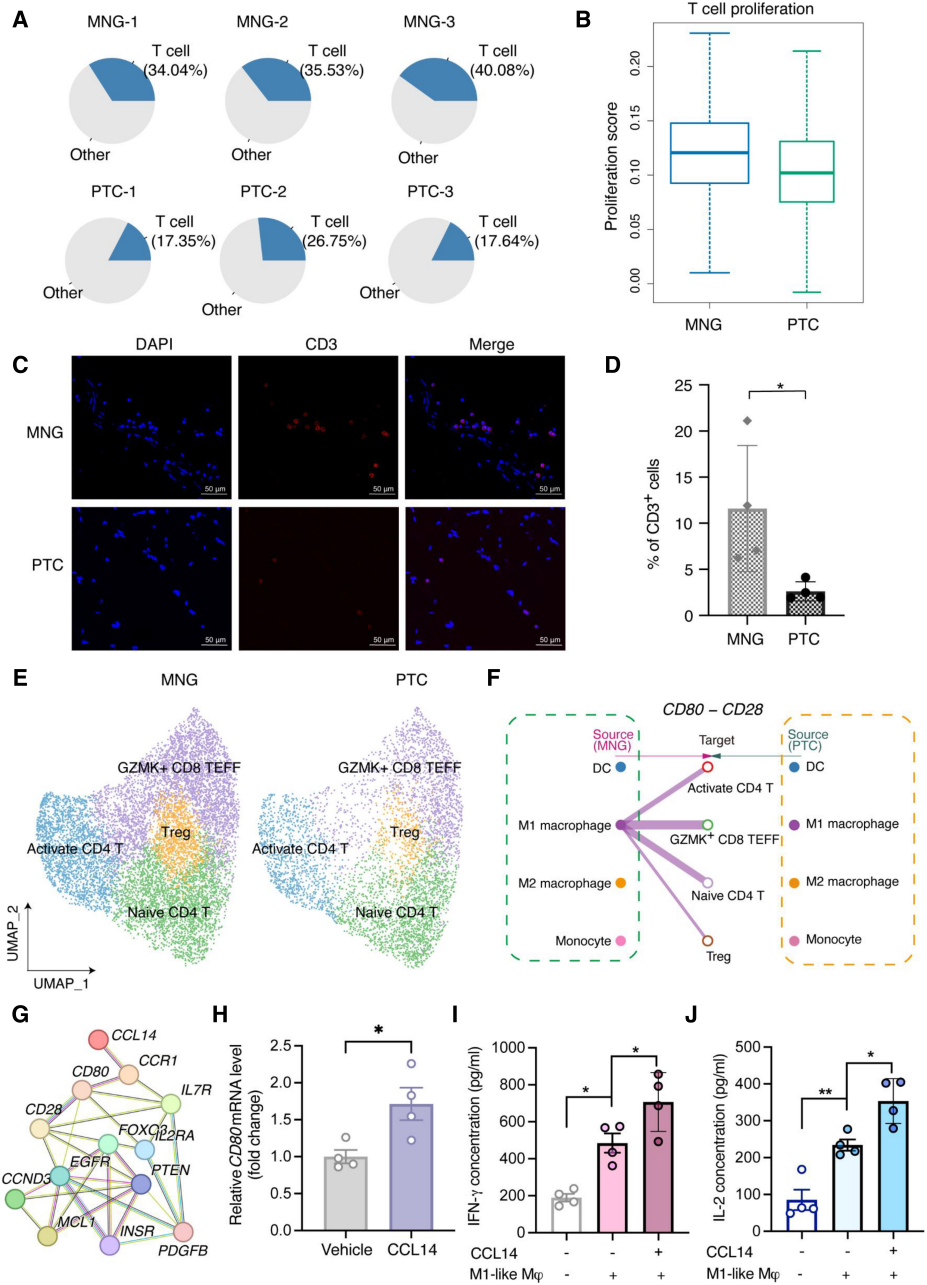
CCL14 reduces the T cell numbers in PAT through the CD80–CD28 signal axis. (A) The pie chart illustrates the proportion of T cells in the total cellular composition of PAT from patients with MNG and PTC, quantified by snRNA-seq. (B) Box plot comparing the “T cell proliferation score”, a gene signature derived from the expression of proliferation-related genes in T cells isolated from PAT of MNG versus PTC patients. (C) Representative immunofluorescence staining images of PAT tissue sections from MNG and PTC patients, stained for the pan-T cell marker CD3 (shown in red) and DAPI (blue) for nuclei. (D) Quantitative analysis of the immunofluorescence staining shown in (C) (*n* = 4). (E) UMAP plot of T cell subclusters, colored by cell type and split by patient diagnosis (MNG versus PTC). (F) Predicted interaction strength of the CD80–CD28 receptor–ligand pair between myeloid cell subclusters (expressing CD80) and T cell subclusters (expressing CD28) in PAT. (G) Protein–protein interaction among CCL14–CCR1, PI3K–AKT signaling, and CD80–CD28 protein network. (H to J) Human M1-like macrophages were treated with recombinant CCL14 and subsequently cocultured with allogeneic T cells to assess macrophage CD80 expression and T cell-stimulatory capacity. (H) *CD80* mRNA expression in macrophages. (I and J) Macrophages treated with CCL14 enhance T cell activation, as reflected by increased secretion of IFN-γ (I) and IL-2 (J) in the coculture supernatants. *n* = 4. Data are presented as means ± SEM. *P < 0.05, **P < 0.01.

Effective T cell activation requires not only antigen presentation but also a critical second signal delivered through the CD80–CD28 costimulatory pathway. Given the marked down-regulation of *CD80* expression in M1-like macrophages, we hypothesized that a deficiency in this costimulatory axis contributes to T cell dysfunction within the PAT microenvironment of PTC patients. Multiple lines of evidence support substantial T cell impairment in PTC-associated PAT. We observed a consistent decrease in the overall proportion of T cells in PTC compared to MNG patients (Fig. 9A), accompanied by a significantly reduced T cell proliferation score (Fig. 9B). Immunofluorescence staining for CD3 further confirmed diminished T cell infiltration in PTC PAT (Fig. 9C and D).

Single-cell stratification of T cells identified 4 major subsets—naïve T cells, activated CD4^+^ T cells, GZMK^+^ CD8^+^ effector T cells, and regulatory T cells (Tregs)—each of which was generally reduced in PTC patients (Fig. 9E). Cell–cell communication analysis revealed strikingly impaired CD80–CD28 interactions between M1-like macrophages and all T cell subsets in PTC, whereas this costimulatory signaling remained robust in MNG PAT (Fig. 9F). Consistent with these findings, T cell subpopulations, particularly activated CD4^+^ T cells, naïve CD4^+^ T cells, and GZMK^+^ CD8^+^ effector T cells, exhibited significant down-regulation of pathways involved in T cell differentiation and immune response activation (Fig. S8A to D). A comprehensive protein–protein interaction network integrated these results, linking CCL14–CCR1 signaling through the PI3K–AKT pathway to CD80 expression and ultimately CD28-mediated T cell activation (Fig. 9G).

To functionally validate the role of the CCL14–CCR1 axis in macrophage–T cell crosstalk, we treated human M1-like macrophages with recombinant CCL14 in vitro, leading to a modest but statistically significant increase in *CD80* mRNA compared with the vehicle control (Fig. 9H). Furthermore, macrophage–T cell coculture experiment was performed to assess the functional consequence of CCL14 treatment on T cell activation. CCL14-treated macrophages exhibited an enhanced ability to stimulate T cells, as demonstrated by increased secretion of interferon-γ (IFN-γ) and interleukin-2 (IL-2) in the culture supernatant (Fig. 9I and J), suggesting that CCL14 augments macrophage-mediated T cell activation, consistent with improved costimulatory signaling.

In summary, our findings demonstrate that in PTC patients, reduced CCL14 secretion from OXPHOS-Ad disrupts CCR1-mediated PI3K–AKT signaling in M1-like macrophages, leading to diminished CD80 expression. The subsequent loss of CD80–CD28 costimulation across T cell subsets results in impaired T cell proliferation and recruitment, revealing a novel mechanism by which PAT microenvironment remodeling promotes immunosuppression in thyroid cancer.

## Discussion

PAT, once regarded merely as an anatomical structure providing mechanical support to the thyroid gland, is now being proposed as a potential paracrine organ that may influence thyroid physiology and pathophysiology. A close anatomical and functional unit exists between the thyroid gland and the PAT. The thyroid is encapsulated by PAT, with only a thin fibrous layer dividing them, and both are interlaced with a shared vascular and neural plexus. This structural integration facilitates a rapid, high-concentration exchange of signaling molecules, creating a unique paracrine signaling niche for thyroid gland. However, while PAT has been implicated in thyroid pathology, fundamental questions regarding its cellular composition, functional specialization, and specific roles in thyroid cancer progression have remained largely unanswered.

**Fig. 10.**
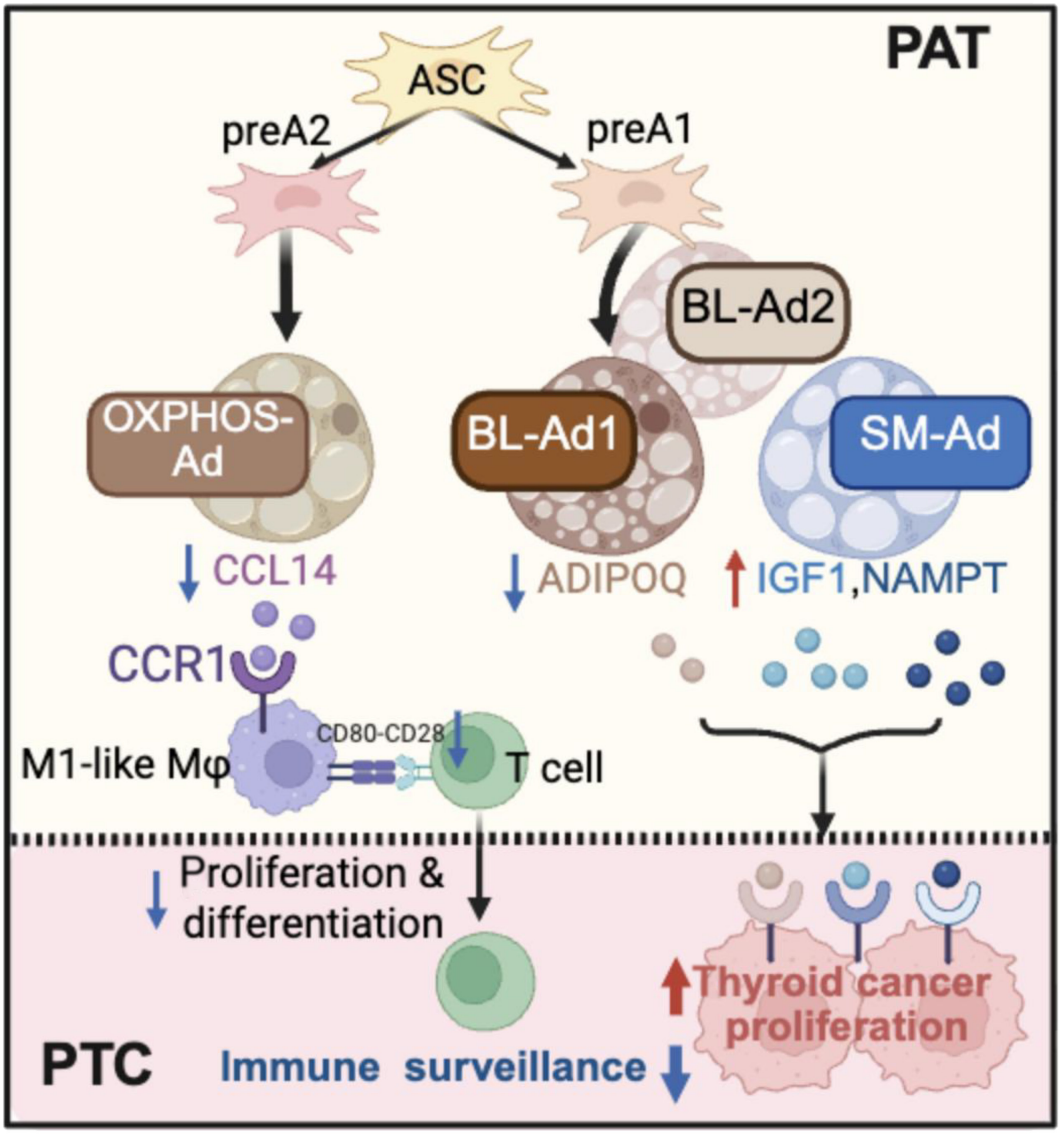
Summary of the mechanisms by which PAT drives thyroid tumorigenesis through adipokine signaling and immune suppression. A high-resolution cellular atlas of human PAT was constructed using snRNA-seq, leading to the identification of multiple heterogeneous adipocyte subsets, including distinct thermogenic adipocyte subpopulations such as BL-Ad1, BL-Ad2, and OXPHOS-Ad, each with independent progenitor cell lineages. In the PAT of patients with PTC, the expression of the tumor suppressor factor ADIPOQ mainly derived from the BL-Ad1 subset is lost, while the expression of the pro-carcinogenic factors NAMPT and IGF1 mainly derived from the SM-Ad subset is up-regulated, collectively promoting the proliferation of thyroid cancer cells. Meanwhile, the chemokine CCL14 is down-regulated in OXPHOS-Ad thermogenic adipocytes of PTC origin, which weakens the CCL14–CCR1 signaling crosstalk between these adipocytes and M1-type macrophages. This leads to reduced CD80 expression in M1-type macrophages, disrupting the CD80–CD28 costimulatory axis and ultimately impairing the proliferation and recruitment capacity of T cells in the PAT of PTC patients.

This study presents the first comprehensive snRNA-seq atlas of human PAT, revealing its intricate cellular landscape and establishing its functional significance in the thyroid tumor microenvironment. Our findings illuminate PAT not as a passive anatomical structure but as a dynamic endocrine and immunomodulatory organ that undergoes substantial remodeling in PTC. Through multi-dimensional analyses, we have delineated distinct nature of PAT compared to other adipose depots, its role in shaping a local immunosuppressive niche, the dual functions of key adipokines in tumor cell proliferation, and the discovery of thermogenic adipocyte subpopulations with potential antitumor functions (Fig. 10 and Table S2).

Our study reveals the unique cellular ecosystem of PAT. While PAT shares a similar adipocyte proportion with SAT and VAT [34], suggesting conservation of core adipocyte lineage characteristics, it is notably enriched in immune cells. PAT contains higher percentage of T cells and B cells, a profile that closely resembles deep neck fat according to an snRNA-seq-based study [36], but differs remarkably from other WATs where immune cells are less abundant [34]. This immune-rich phenotype implies that PAT may function as a regional immune hub, a feature rarely observed in other adipose depots and one that likely stems from its direct proximity to thyroid tissues. Furthermore, PAT harbors specialized adipocyte subpopulations that have not been widely reported in other adipose depots, including IR-Ad enriched in T/B cell receptor signaling pathways, SM-Ad, and distinct thermogenic subpopulations.

The discovery of thermogenic adipocyte subpopulations within PAT represents a particularly intriguing finding, challenging the conventional characterization of this depot as primarily WAT. We identified 2 distinct brown-like adipocyte subpopulations (BL-Ad1 and BL-Ad2) showing striking homology to the established brown adipocyte populations H-Ad-1 and H-Ad-5 in human deep neck fat, along with an OXPHOS-dependent thermogenic subpopulation (OXPHOS-Ad). Although these subpopulations exhibit characteristic thermogenic features, they express surprisingly low levels of UCP1, the classical mediator of nonshivering thermogenesis [24]. Instead, these adipocytes are enriched in genes associated with various futile cycles [25], indicating that they likely achieve thermogenesis through noncanonical, UCP1-independent pathways previously documented in other human fat depots [26]. Interestingly, we found that different adipocyte subpopulations employ distinct futile cycle mechanisms, wherein OXPHOS-Ad is specifically enriched for genes involved in creatine and Ca^2+^ cycles, whereas BL-Ad1 shows enrichment in lipid cycling genes. This differential enrichment suggests specialized functional roles and modes of action among these thermogenic adipocytes. This mechanistic divergence is further underscored by their differential expression of thyroid hormone receptor isoforms, highlighting distinct regulatory programs. This is of particular relevance given the established role of thyroid hormones in activating brown and brown-like adipocytes [49,50]. These findings not only represent the first report of thermogenic adipocytes in PAT but also open new avenues for investigating their specialized contributions to thyroid pathophysiology and energy metabolism. However direct functional validation [e.g., Seahorse-based oxygen consumption rate (OCR)/extracellular acidification rate (ECAR) measurement] remains technically challenging due to the limited yield of viable mature adipocytes from human PAT and the difficulty of isolating intact OXPHOS-Ad cells. Future studies using advanced approaches, such as single-cell metabolic profiling or genetic models, will be required to definitively establish their thermogenic capacity.

The current study also employed developmental trajectory analysis to elucidate the origin of adipocyte functional specialization, revealing that distinct thermogenic subpopulations originate from separate progenitor pools via 2 independent differentiation pathways. Specifically, preA1 progenitors give rise to the BL-Ad1/BL-Ad2 lineage, while preA2 precursors differentiate exclusively into OXPHOS-Ad cells. This developmental divergence, coupled with their distinct mechanistic programs and receptor expression profiles, establishes the PAT as a previously unrecognized site of specialized thermogenic activity, potentially critical for localized thermoregulation, energy metabolism, and thyroid homeostasis. Adipocyte precursor cells (APCs) are a heterogeneous population [51–53]. Among them, Dpp4 + ASC exhibits enhanced proliferative capacity and multilineage potential, whereas Icam1+ preA is highly committed to the adipose lineage and undergo rapid differentiation with minimal stimulus [51]. However, under specific conditions such as aging and high-fat diet-induced obesity, ASCs and other APC subclusters can also contribute directly to adipogenesis [54,55]. In our study, we observed no significant differences in these developmental trajectories between MNG and PTC conditions (data not shown). Nevertheless, given the thermogenic nature of the BL-Ad1/2 and OXPHOS-Ad adipocytes, a key future direction will be to investigate whether de novo generation of these specialized adipocytes from other APC clusters is modulated under specific pathological conditions, such as obesity, thyroid disease, and aging.

ETE represents a well-established poor prognostic factor in PTC [30,33], suggesting potential involvement of the PAT in tumor progression. While clinical studies have implicated adipokine dysregulation in thyroid cancer pathogenesis, such as the documented loss of the antitumor adipokine ADIPOQ [20,56,57] and elevated levels of mitogenic factors like IGF1 [58,59] and NAMPT [60,61] in PTC tissues, direct functional evidence for the role of PAT has remained limited. In this study, we provide the first direct demonstration that PAT actively contributes to PTC progression via a pathogenic adipokine triad. Through integrated machine learning and cell–cell interaction analysis, we identified a PTC-specific secretory signature in PAT characterized by loss of ADIPOQ and concomitant gain of NAMPT and IGF1. This triad functionally underlies the tumor-promoting capacity of the PAT secretome, which enhanced thyroid cancer cell proliferation, DNA synthesis, and colony formation, effects that were consistently more pronounced in PTC-derived PAT than in PAT from MNG controls. Crucially, we validated this mechanism by showing that restoration of ADIPOQ signaling, combined with inhibition of NAMPT and IGF1 pathways, effectively blunts PAT-induced tumor proliferation. These findings not only corroborate prior clinical observations, such as the inverse correlation between circulating ADIPOQ and thyroid cancer risk and the association of elevated IGF1 and NAMPT with advanced disease, but also extend them by establishing a causal link between PAT-derived adipokine imbalance and PTC progression, thereby positioning PAT as a dynamic tumor-adipose microenvironment (TAME) and a potential target for adjuvant therapy.

Apart from the direct impact of PAT-originated adipokines on thyrocytes**,** PAT-derived factors also exert their modulatory function on other cell types in thyroid microenvironment. We identified significant down-regulation of *CCL14* in the OXPHOS-Ad subpopulation in PTC patients at both transcriptomic and proteomic levels. CCL14 belongs to the CC chemokine family, which is considered as a chemoattractant of monocytes, eosinophils, T lymphoblasts, and neutrophils [62]. Consistent with our finding, Zhang et al. [63] reported that CCl14 expression in thyroid cancer tissues is down-regulated compared with normal tissues. Patients with low expression of CCL14 had significantly lower rates of disease-free interval, progression-free interval, and low immune infiltrates [63], implying the potential effect of CCL14 on the immune response and immune therapy in thyroid cancers. CCR1, CCR5, and a weak affinity receptor CCR3 are the receptors of CCL14 [46]. In PAT, CCR1 represents the predominant receptor for CCL14, with a particular enrichment in M1-like macrophages. Furthermore, CCL14 down-regulation is associated with a disrupted CCR1-mediated PI3K–AKT–FOXO3 signaling in M1-like macrophages, leading to impaired CD80 expression. The consequent loss of the critical CD80–CD28 costimulatory signal between M1-like macrophages and T cells likely silences T cell activation and proliferation, which is experimentally validated in PAT specimens in this study. This functional relevance of the CCL14–CCR1–CD80 axis in regulating T cell immunity provides a link between PAT microenvironment alterations and the impaired antitumor immune surveillance in PTC (Fig. 10). Furthermore, our study unveils a potential connection between thermogenic adipocyte (OXPHOS-Ad) function and immune regulation, in that a thermogenically active PAT may help sustain a local immune-active microenvironment. This perspective aligns with growing evidence of the intricate connections between metabolism and immunity in the tumor microenvironment [64], although the detailed mechanism and pathological implications warrant future investigations.

ADIPOQ, NAMPT, IGF1, and CCL14 are known to be dysregulated in obesity and insulin resistance [65–69]. It is therefore plausible that metabolic disorders could amplify or modify the adipokine imbalance that we observed in PAT, potentially exacerbating tumor-promoting signaling or altering immune crosstalk within the microenvironment. Furthermore, systemic metabolic inflammation may also introduce additional layers of regulation involving immune cells and stromal compartments [70,71], which warrants future studies. Future studies incorporating patients with defined metabolic phenotypes (e.g., obesity and insulin resistance) will be essential to determine how metabolic status reshapes the PAT secretome and whether the identified adipokine triad is conserved, enhanced, or altered under these conditions.

Another limitation of our study is the relatively low sample size, which requires expansion in future studies for broader generalizations. However, no publicly available transcriptomic datasets currently exist for human PAT, precluding independent validation using external datasets. Future studies incorporating larger cohorts and independent PAT datasets will be essential to further validate the reproducibility and clinical relevance of these findings. Furthermore, the precise regulation of CCL14 transcription and secretion from OXPHOS-Ad remain to be fully elucidated. Future studies investigating the direct link between thermogenic activity and CCL14 secretion will be crucial to fully understand this intriguing connection. Importantly, while our in vivo and in vitro data support a tumor-promoting role of PAT, the current study is limited to tumor growth endpoints and does not address its impact on recurrence, metastasis, or overall survival.

In conclusion, our comprehensive characterization of PAT reveals it as an active functional unit within the thyroid tumor microenvironment that influences cancer progression through both direct effects on tumor cells and modulation of the immune landscape. The discovery of distinct adipocyte subpopulations, their developmental trajectories, and their specific roles in tumor promotion and immune regulation opens new avenues for understanding thyroid cancer biology and developing targeted interventions. These findings position PAT as a critical and previously underappreciated player in thyroid cancer pathophysiology, offering new insights into the complex interplay between metabolism, immunity, and cancer in the thyroid microenvironment.

## Methods

### Clinical sample collection

The PAT was collected from Guangzhou Huaqiao Hospital affiliated to Jinan University and approved by the ethics committee (IIT-2024-741). Female adult patients with either PTC at T1aN0M0 I stage or MNG were recruited for this project. Those with metabolic disorders, including diabetes, were excluded. During the surgery, the PAT located near the central lymph nodes in the lower pole of the thyroid gland was collected, and then either snap frozen in liquid nitrogen or fixed in 4% formalin in phosphate-buffered saline (PBS) for future use. Alternatively, the PAT was freshly isolated and used for CM collection. Serum samples were collected from 75 participants, including 24 healthy controls and 51 patients categorized as having goiter (*n* = 12), low-risk (*n* = 18), or high-risk (*n* = 21) differentiated PTC. Written informed consent was obtained from all individuals. After a 12-h overnight fast, blood was collected from all subjects, and the plasma was transported on ice within 1 h, separated by 2 successive centrifugations (1,880*g*, 10 min and 2,500*g*, 10 min) to obtain platelet-free plasma, and stored at −80 °C until analysis.

### Collection of PAT CM

Human PAT was freshly isolated, rinsed once in PBS, and cut into smaller pieces of approximately 1 mm^3^. The tissues were then cultured in Dulbecco’s modified Eagle’s medium (DMEM high glucose, Gibco) at 100 mg/ml and incubated for 24 h at 37 °C in an environment with 5% CO₂. The CM was filtered through a 0.22-μm filter (Minisart High Flow) before storage at −80 °C for future use.

### Cell nuclei extraction and cDNA library construction

PAT (200 mg) was used to isolate cell nuclei. Samples were kept frozen on dry ice until nuclei were isolated, and all sample processing steps were performed on ice. The cell nuclei of PAT were extracted using a commercial kit (52201-10, SHBIO), followed by mixing with water-in-oil microreactors for complementary DNA (cDNA) amplification and library construction using the SeekOneDD Single Cell 3′ Transcriptome Kit following the protocol. Paired-end sequencing of libraries was performed using the Illumina NovaSeq 6000 system.

### Data processing of snRNA-seq data

The raw sequencing data in FastQ format were aligned to a pre-built GRCh38 reference database and counted using CellRanger 7.2.0 with default parameters. The processed files were then imported into the R package Seurat 5.0.3 for quality control. Nuclei with less than 200 or more than 8,000 features or more than 20% of mitochondrial genes were defined as low-quality nuclei and filtered out. After filtering, the median number of unique molecular identifier (UMI) detected per cell was 2,911 and the median number of genes detected per cell was 1,640.

### Integration, clustering, and annotation

The integration and clustering analysis were performed using the R package Seurat 5.0.3. Firstly, the gene counts were normalized by the total expression for each cell, and a subset of highly variable features was identified for downstream analysis. Secondly, data were scaled prior to dimensional reduction and then principal components analysis (PCA) was performed. Thirdly, the mutual nearest neighbor (MNN) method was used to integrate all the data to eliminate the batch effect. For clustering, the top 20 principal components (PCs) were used to construct a k-nearest neighbors (KNN) graph and a resolution of 1 was used to group cells together. Then, both statistical method t-SNE and Uniform Manifold Approximation and Projection (UMAP) were used for visualization. For annotation, the marker genes of each single cluster were identified by log_2_ fold change more than 1, then tested by feature and violin plots, and manually selected. Clusters were annotated based on the classical cell type marker genes and markers of each cluster, including adipocyte (*ADIPOQ*), ASPC (*PDGFRA*), B cell (*CD79A*), endothelial cell (*PECAM1*), LEC (*PROX1*), myeloid cell (*MAFB*), neutrophil (*CSF3R*), pericyte (*STEAP4*), SMC (*MYOCD*), and T cell (*CD3D*). The subcluster of cell populations was also used in the above methods, and the annotation was according to classical marker genes and markers in each subpopulation.

### Functional class scoring and gene set scoring

The differential gene sets enriched in cell populations were calculated by R package irGSEA. The reference gene set was the predefined Kyoto Encyclopedia of Genes and Genomes (KEGG) gene set in the MSigDB database. AUCell and Ucell were used for score calculation. The kernel density was estimated using Gaussian distribution. The brown score in adipocyte subpopulations was calculated by AddModuleScore() function in R package Seurat 5.0.3, and 25 browning-related genes (*ADIPOQ*, *CA4*, *CD36*, *CIDEA*, *CITED1*, *DIO2*, *ELOVL3*, *EPSTI1*, *FABP4*, *HOXC9*, *IRF4*, *KCNK3*, *HX8*, *MTUS1*, *PDK4*, *PLIN1*, *PPARG*, *PPARGC1A*, *PRDM16*, *SHOX2*, *SLC2A4*, *TMEM26*, *TNFRSF9*, *UCP1*, and *ZIC1*) were used for calculation [40].

### Trajectory analysis

The Monocle 2.30.1 package was used for trajectory analysis to identify the progenitor cells of adipocyte subpopulations. Ordered genes based on mean expression level that were expressed in more than 10 cells were retained. Then, the data were reduced in dimensionality using the reverse graph embedding (DDRTree) algorithm with default parameters. According to the expression trend of the order gene, the cells were sorted and the trajectory was constructed. The differentialGeneTest() function was used to identify pseudotime-related genes, and the branched expression analysis modeling (BEAM) was performed to find genes that are regulated in a branching-dependent manner.

### RNA velocity

RNA velocity was performed using scVelo with default parameters [72]. The count matrices were size normalized to the median of total molecules across cells. The top 2,000 highly variable genes were selected for spliced and unspliced mRNA. For velocity estimation, first- and second-order moments were computed for each cell across its 30 nearest neighbors.

### DEG analysis

DEG analysis was conducted using DESeq2 (version 4.0.0) package in R (version 4.0.0), with the filtering threshold set at a fold change (FC) >1.5 and the FDR adjusted *P* value <0.05.

### GO and KEGG enrichment

GO and KEGG pathway analysis were performed using clusterProfiler (version 4.10.1) package in R. Genes were filtered to have Benjamini–Hochberg corrected *P* value < 0.05 and then evaluated for enrichment in GO biological pathways or KEGG pathways.

### Analysis of cell–cell interactions

The R package CellChat 1.6.1 was used to analyze the interactions between different cell populations [73]. The CellChatDB contains databases of human and mouse literature supporting ligand–receptor interactions. Communication probabilities were calculated, and cellular communication networks were inferred by calculating the average gene expression per cell population.

### Machine learning-based screening for secreted factors

To identify key secretory factors, DEGs derived from adipocyte subclusters were initially cross-referenced with the secretory factor database from the CellChat package (v1.6.1) to obtain a list of differentially secreted factors. Cells expressing at least 30% of these genes were retained for subsequent analysis.

Feature selection was then performed to screen for genes that contributed significantly to the model. LOPO-CV was applied throughout the study to eliminate the interference of individual differences on model stability and result reliability. In each validation round, samples from one patient were set as the test set, and data from all remaining patients were used for model training. Validation was completed after cycling through all samples, and model performance was evaluated based on the average AUC value. Moreover, 10-fold cross-validation was adopted to improve the stability of feature ranking. The dataset was randomly divided into 10 subsets. Nine subsets were used as the training set to establish the XGBoost model, and the remaining one served as the test set. Feature importance was assessed by calculating SHAP values. The process was repeated 10 times to ensure that each subset was used as the test set in turn. Retained genes were ranked according to average SHAP values.

RFE was performed to gradually remove low-ranked features. The model was reconstructed, and the AUC value was calculated after each elimination until the average AUC no longer increased. The gene combination corresponding to the maximum AUC value was identified as the optimal gene set capable of distinguishing PTC from MNG cells, suggesting their potential involvement in thyroid cancer progression. In addition, permutation tests were conducted to further verify that the gene signature was resistant to individual variation and free from sample bias. Finally, the interaction probability between the selected ligand genes expressed in PAT and their corresponding receptors on thyroid cells was computed, enabling the identification of secreted factors with the highest likelihood of directly influencing thyroid cells.

### Comparison among PAT, SAT, VAT, and deep-neck fat

The published single-cell RNA sequencing (scRNA-seq) and snRNA-seq datasets of human SAT and VAT were from Emont et al. [34] [Single Cell Portal (study no. SCP1376)], and the scRNA-seq data of human deep neck adipose tissue were from Sun et al. [36] (E-MTAB-8564). To compare the abundance of various cell clusters among different datasets, the number of cell clusters was divided by the total number of cells to obtain the relative percentage of each cluster. To calculate the correlations between cell clusters in PAT and other datasets, the count matrix was extracted, the mean of the genes was calculated, and then the cor() function in the stats package (version 4.3.2) was used to calculate the correlation score with the Pearson method.

### Analysis of the thyroid cancer data

The thyrocytes in female patients were extracted from the scRNA-seq dataset of human PTC samples (GSE184362) [43] for further analysis. The integration, clustering, and annotation of the data were performed as described above. The thyrocytes were divided into normal, pre-malignant, and malignant subtypes according to the method described in the paper [43]. The mRNA levels of 13 genes (*TG*, *TPO*, *SLC26A4*, *DIO2*, *TSHR*, *PAX8*, *DUOX1*, *DUOX2*, *NKX2-1*, *GLIS3*, *FOXE1*, *TFF3*, and *FHL1*) were log-normalized and scaled. TDS was calculated using these 13 genes.

### Xenograft mouse model and adipokine manipulation

All animal procedures were approved by the Institutional Ethics Committee of the First Affiliated Hospital of Sun Yat-sen University ([2026]058) and conducted in accordance with relevant guidelines. Four-week-old female nude mice were obtained from the Animal Center of Sun Yat-sen University and maintained under specific pathogen-free conditions. To recapitulate the adipokine profile observed in benign MNG, mice were randomly assigned to 2 groups. A recombinant AAV vector was engineered to simultaneously overexpress mouse *Adipoq* under the control of the CAG promoter, together with U6 promoter-driven short hairpin RNAs (shRNAs) targeting mouse *Igf1* and mouse *Nampt* (triad modulation). The construct was generated using pAAV-CAG-GFP (green fluorescent protein) (Addgene #37825) as the backbone. AAV particles were locally injected into the inguinal subcutaneous fat pad at a dose of 1 × 10^10^ viral genome copies per fat pad. Seven days after AAV administration, human PTC cells [BCPAP; 5 × 10^6^ cells suspended in 100 μl of PBS mixed with 50 μl of Matrigel (Corning, 356234)] were subcutaneously implanted adjacent to the injected fat pad to establish xenograft tumors. Tumor growth was monitored periodically by caliper measurements, and tumor volume was calculated accordingly. At the experimental endpoint, tumors were excised and weighed.

### Proteomics analysis of PAT CM

The PAT CM was subjected to liquid chromatography–tandem mass spectrometry (LC-MS/MS) analysis. The main procedures included protein extraction, peptide enzymatic hydrolysis, LC-MS/MS data-independent acquisition (DIA), database search, qualitative and quantitative result analysis, and bioinformatics analysis. All samples were subjected to trypsin digestion using the filter-aided proteome preparation (FASP) method. The digested peptides of the samples were desalted using a C18 cartridge. After lyophilization, the peptides were reconstituted by adding 40 μl of 0.1% formic acid solution, and the peptide concentration was determined by measuring the absorbance at 280 nm (A280). An appropriate amount of indexed retention time (iRT) standard peptides was added to the digested peptides of each sample, and DIA mass spectrometry detection was performed using an Astral high-resolution mass spectrometer. The DIA data were processed using DIA-NN software. The software parameters were set as follows: enzyme was trypsin, max miss cleavage site was 1, fixed modification was carbamidomethyl (C), and dynamic modifications were set as oxidation (M) and acetyl (protein N-term). The proteins identified by database search must pass the set filtering parameter of FDR < 1%.

The obtained protein quantitative data were first subjected to PLS-DA analysis using the R package “ropls” to evaluate the aggregation degree of samples within groups and the differential points between groups. Subsequently, the “getVipVn()” function was used to calculate the VIP values of proteins, and proteins with a VIP value greater than 1 were selected as differential metabolites. Those proteins with the FC value > 1.5 and the *P* value < 0.05 were significant.

### Cell culture

The human PTC cell line BCPAP was purchased from Cytion. The cells were cultured in RPMI 1640 (Gibco) supplemented with 10% fetal bovine serum (FBS; Gibco) and 1% penicillin–streptomycin at 37 °C with 5% CO₂. When the cell confluency reached 80%, the cells were washed with PBS and then detached using TrypLE Select Enzyme (Gibco25200072).

### Cell proliferation assays

Cells were seeded in 96-well plate at 1,000 to 5,000 cells per well and cultured in human PAT CM (1:3 diluted in RPMI 1640) containing 10% FBS. Cell viability was measured using CCK-8 assay kit (Dojindo, Japan, #CK04). Serum-free medium (100 μl) and CCK-8 reagent (10 μl) were added to each well. The cells were incubated at 37 °C for 3 h in the dark, and the light absorption at 450 nm was measured at 0, 24, and 48 h using microplate spectrophotometer (Bio-Rad, xMark). For the EdU assay, after 48 h of CM or blank medium treatment, 10 μM EdU (MCE; HY-118411) was added to the culture medium. One hour later, the cells were fixed with 4% formaldehyde in PBS and permeabilized with 0.5% Triton X-100. Subsequently, AFDye azide (MCE, HY-D2177) was applied for 30 min at room temperature under dark condition, followed by nuclear staining with Hoechst 33342 for another 30 min. The cells were imaged using a Ti2E fluorescence microscope and quantified using ImageJ. For the colony formation assay, cells were seeded at 500 cells per well and cultured for 24 d. The medium was changed every 7 d.

### Immunofluorescence staining

The PAT was fixed in 4% formalin in PBS solution for 24 h, followed by dehydration, paraffin embedding, and sectioning into slices with a thickness of 5 μm. After deparaffinization and rehydration, the sections were blocked for 1 h in a PBS solution (pH 7.4) containing 3% bovine serum albumin (BSA; Roche) and 10% FBS. After that, the sections were incubated with the primary antibodies in PBST (PBS supplemented with 0.1% Tween 20) containing 3% BSA overnight at 4 °C. After washing 3 times with PBST, the sections were incubated with fluorophore-conjugated secondary antibodies for 1 h in the dark. The sections were counterstained and mounted using Gold Anti-fade Mountant with 4′,6-diamidino-2-phenylindole (DAPI) (Thermo Fisher Scientific). The images of the immunofluorescence-stained sections were acquired using a Carl Zeiss LSM800 inverted confocal microscope.

### Macrophage–T cell coculture assay

Peripheral blood mononuclear cells (PBMCs) were isolated from healthy donors by density gradient centrifugation, and CD14^+^ monocytes were purified using magnetic bead selection (Miltenyi Biotec). Monocytes (2 × 10^5^ cells/well) were differentiated into macrophages in RPMI 1640 supplemented with 10% FBS and 50 ng/ml macrophage colony-stimulating factor (M-CSF) for 6 d, with medium replacement every other day and recombinant human CCL14 (MCE, 250 ng/ml) or vehicle added. On day 7, macrophages were stimulated with 100 ng/ml lipopolysaccharide (LPS) and 20 ng/ml IFN-γ in the presence or absence of CCL14 for 48 h to induce M1-like polarization. CD80 mRNA level in macrophages was assessed by quantitative PCR. For coculture experiments, CD14^−^ cells from an allogeneic donor were isolated and cocultured with treated macrophages at a 1:5 ratio in U-bottom 96-well plates for 24 h. IFN-γ and IL-2 levels in supernatants were quantified by ELISA (R&D Systems).

### Statistical analysis

The results were presented as means ± standard error of mean (SEM) unless specified otherwise. For cell-based studies, statistical analysis was performed with GraphPad Prism 7. After calculating normality by D’Agostino–Pearson omnibus test, 2-sided unpaired *t* test was used to compare 2 groups of samples. One-way or 2-way analysis of variance (ANOVA) analysis followed by Tukey’s honestly significant difference post hoc test was used for multiple group comparisons. *P* < 0.05 was regarded as significant. Sample size and *P* values are described in the figure legends.

## Data Availability

The authors state that the data supporting the findings of this study are provided in the main article and the Supplementary Materials and may also be obtained from the corresponding authors upon reasonable request.
