# Supplementary material for: Perithyroidal Adipose Tissue Drives Thyroid Tumorigenesis through Adipokine Signaling and Immune Suppression
Source: Research (Wash D C). 2026 Jul 14;9:1360. doi: 10.34133/research.1360 (PMC13365586; doi:10.34133/research.1360)
Supplement: Supplementary 1 — Figs. S1 to S8 Tables S1 and S2 [file research.1360.f1.docx]

**SUPPLEMENTARY MATERIALS**


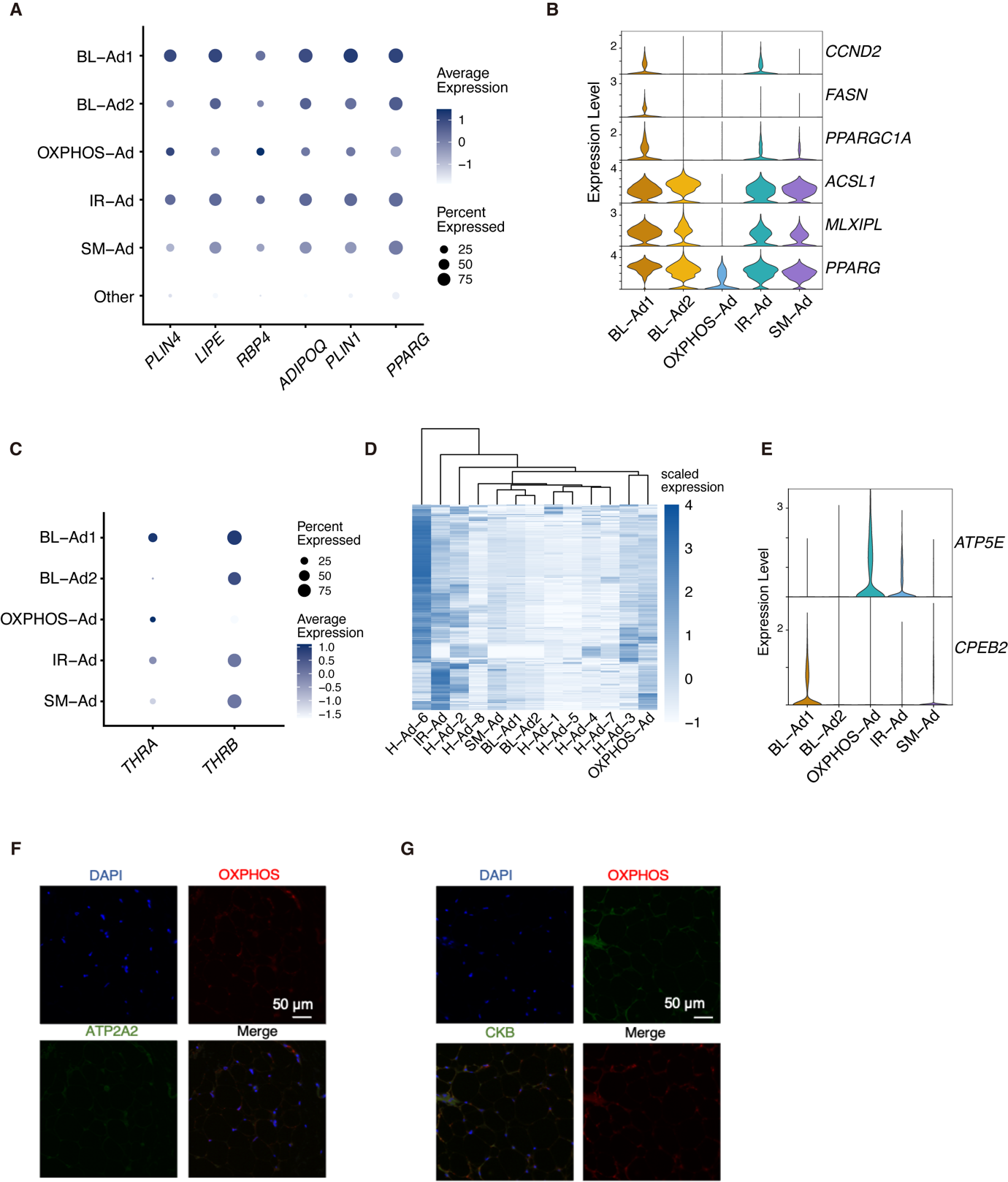
**Supplementary figures**

**Figure S1. The feature of adipocyte subpopulations.** (A) Dot plot illustrating the expression level of established adipocyte marker genes across all identified adipocyte subclusters and non-adipocyte cell from snRNA-seq of human PAT.(B)Violin plots showing the expression distribution of a defined set of canonical target genes of the thyroid hormone receptors THRA and THRB across the adipocyte subpopulations. (C) Dot plot illustrating the expression levels of thyroid hormone receptor genes, THRA and THRB, across the adipocyte subclusters. (D) Alluvial diagram visualizing the transcriptional similarity between adipocyte subclusters identified in PAT and those from deep neck fat. (E) Violin plots showing the distribution of expression levels for ATP5E and CPEB2 across the adipocyte subclusters. (F)-(G) Representative immunofluorescence staining of ATP2A2 (F) and CKB (G) in human PAT at areas of low OXPHOS expression.

**
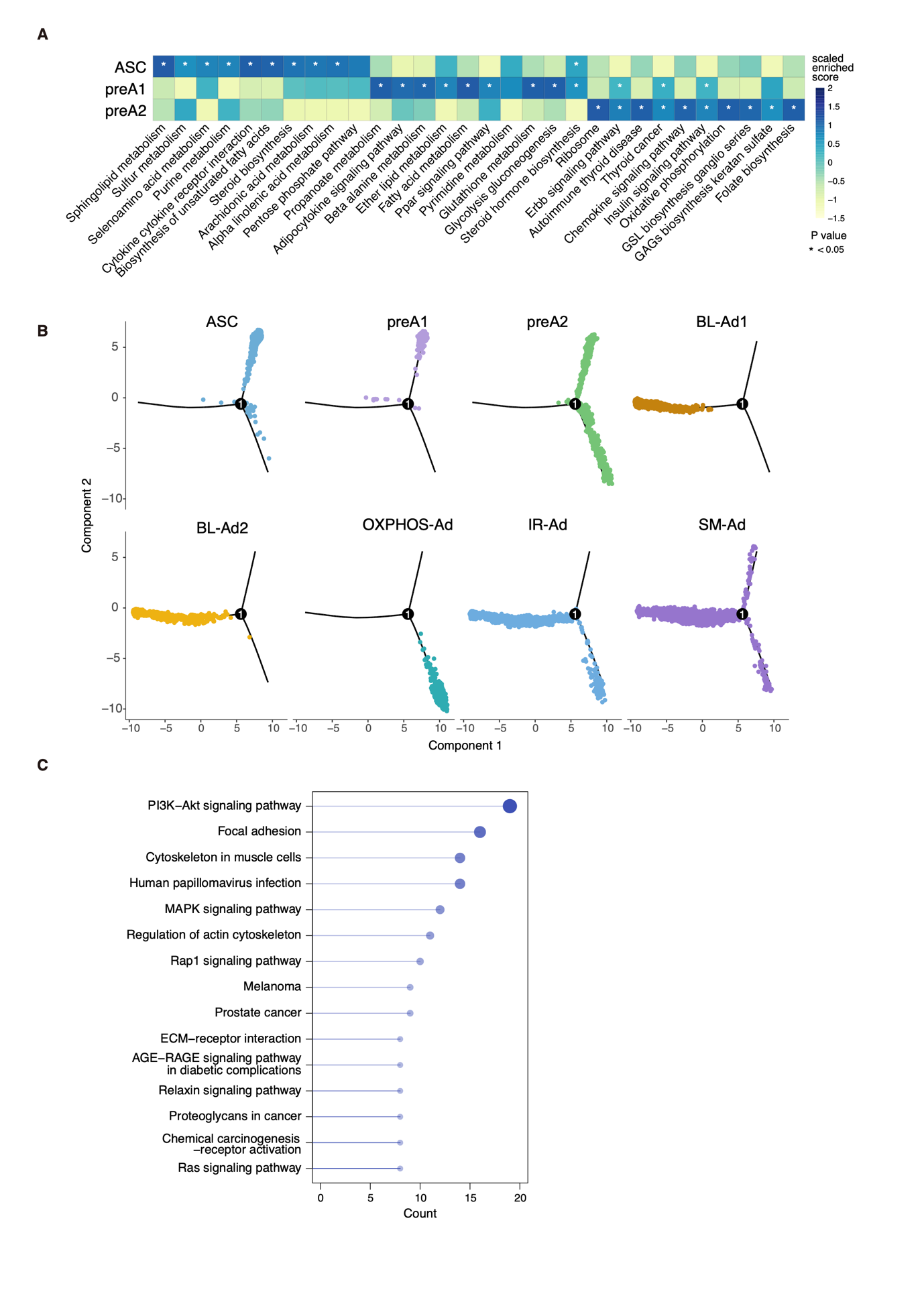
Figure S2. The trajectory among ASPC and adipocyte subclusters.** (A) The results of GSEA in ASPC subclusters. The plot displays the significantly enriched functional gene sets (with P < 0.05) for each subcluster. (B) The trajectory among ASPC and adipocyte subpopulations, which reconstructs the potential developmental paths and branching points during adipocyte differentiation, suggesting distinct lineage relationships. (C) Gene ontology (GO) enrichment of C2 enriched genes in Figure 3G.

**
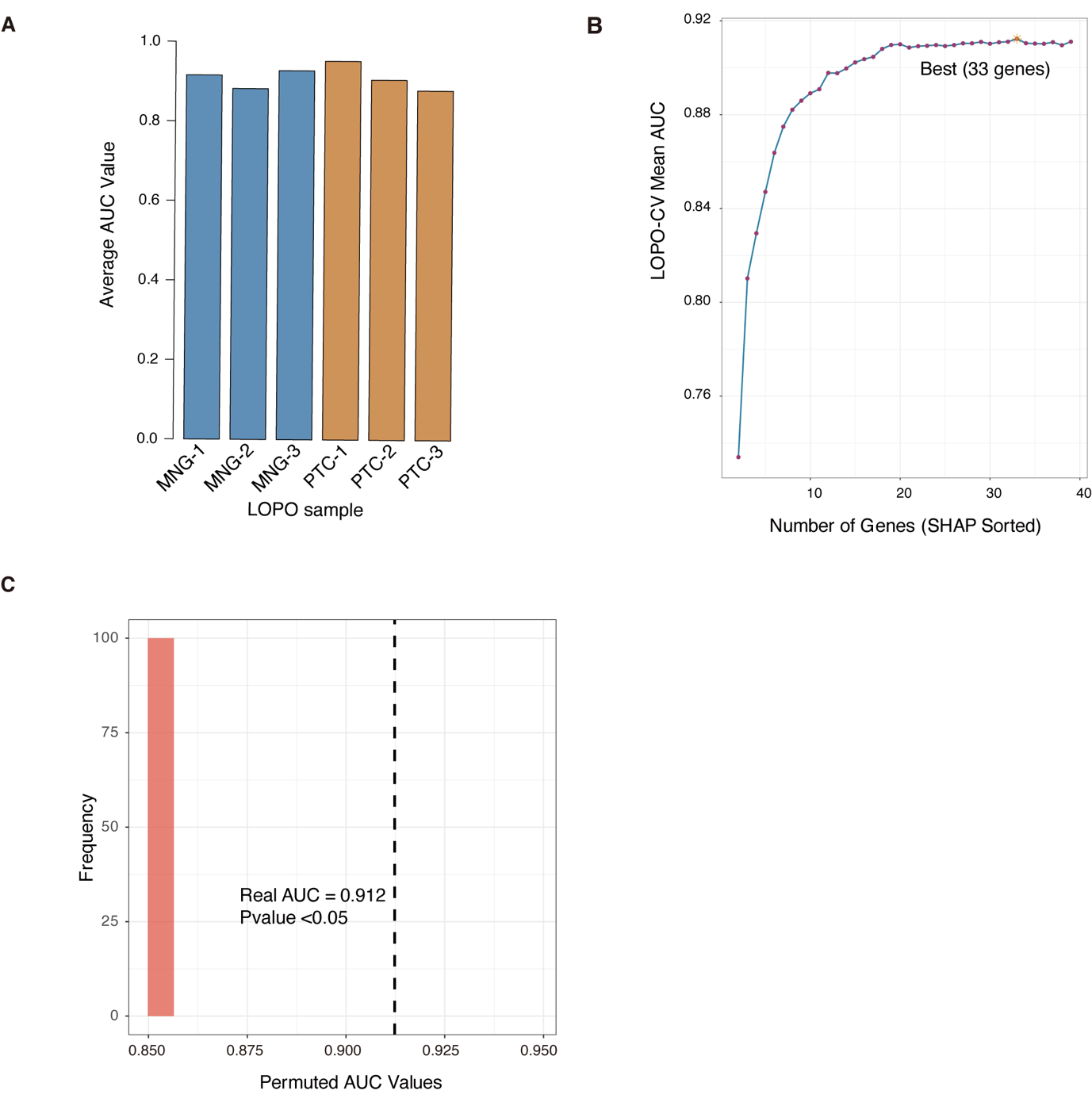
**

**Figure S3. Selection of key secreted factors with the greatest influence on the PTC progression outcome by machine learning**. (A) Bar plot displaying the mean AUC value in each round of LOPO-CV verification. (B) Dot plot shows the predictive performance, measured by AUC, of gene sets comprising the top N ranked secreted factors. (C)Histogram shows the real AUC and random-level AUC for the 33 genes **
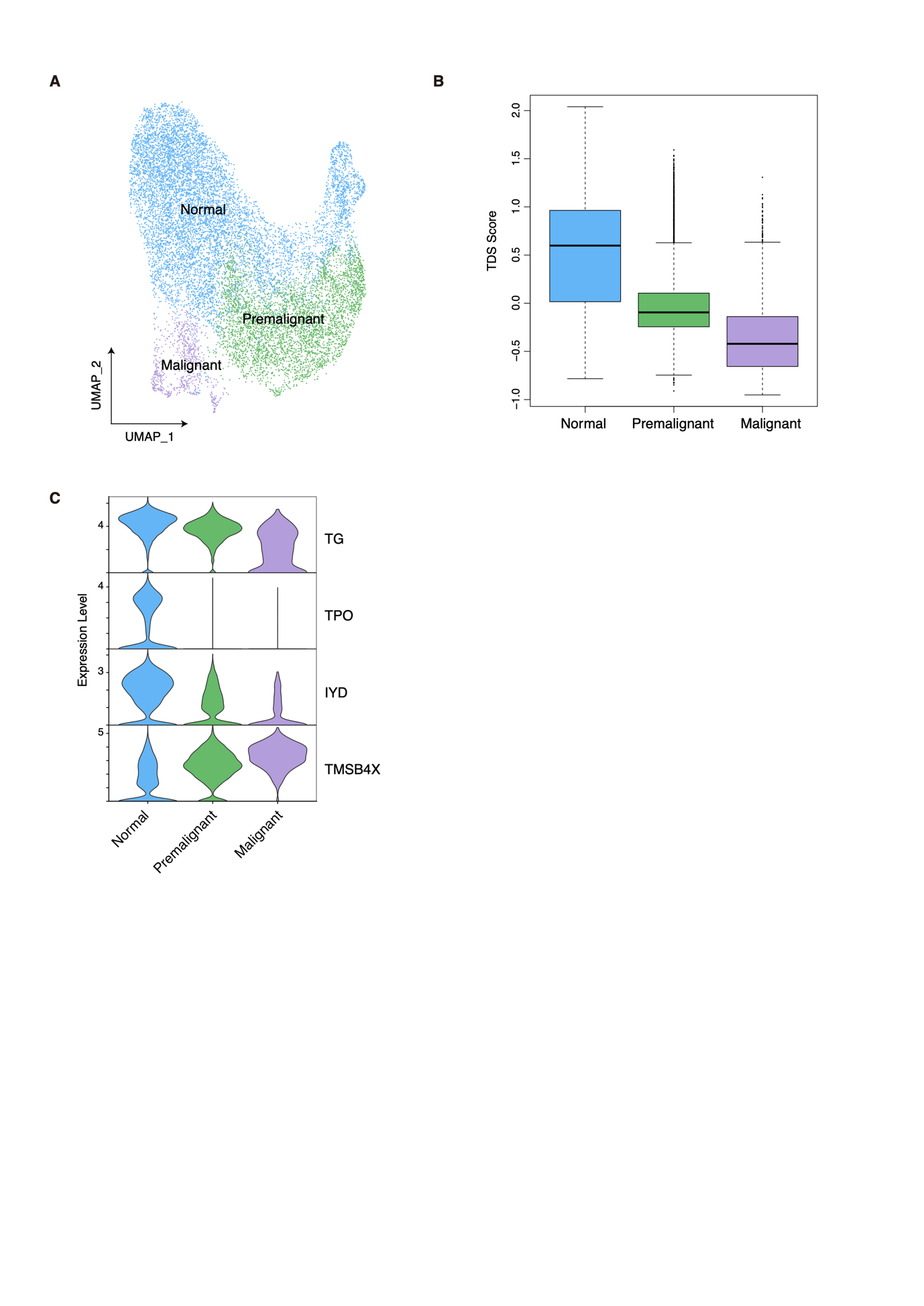
**identified by LOPO-CV.

**Figure S4. Cellular composition of the thyroid tissue.** Single cell RNA-Seq data of human thyroid tissue was re-analyzed. (A) UMAP plot showing the thyrocyte subclusters in thyroid tissue.(B) Violin plots showing the distribution of the "Thyrocyte Differentiation Score (TDS)" across the thyrocyte subclusters.(C) Violin plots comparing the expression of classic thyrocyte functional markers (TG, TPO, IVD) and TMSB4X across the thyrocyte subclusters.

**
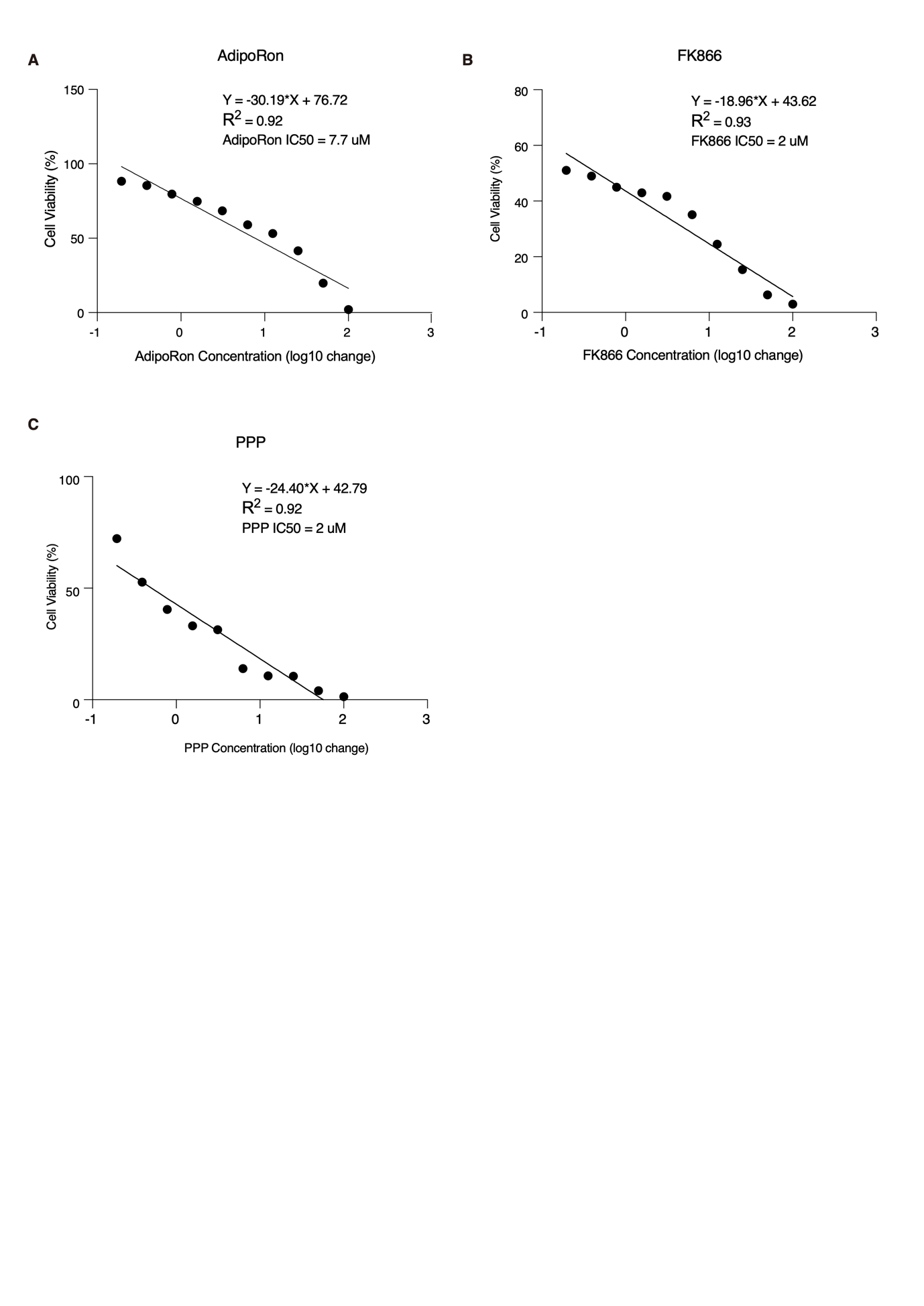
**

**Figure S5. Determination of IC50 for the pharmacological compounds used in the ex vivo study.** (A-C) Dose-response curves and calculated IC50 values for three pharmacological compounds used to treat the BCPAP human thyroid cancer cell line in the functional experiments (Figure 6). Cells were treated with a range of concentrations of each compound, and cell viability was measured using a CCK-8 assay. (A) IC50 determination for AdipoRon. (B) IC50 determination for FK866. (C) IC50 determination for PPP.

**
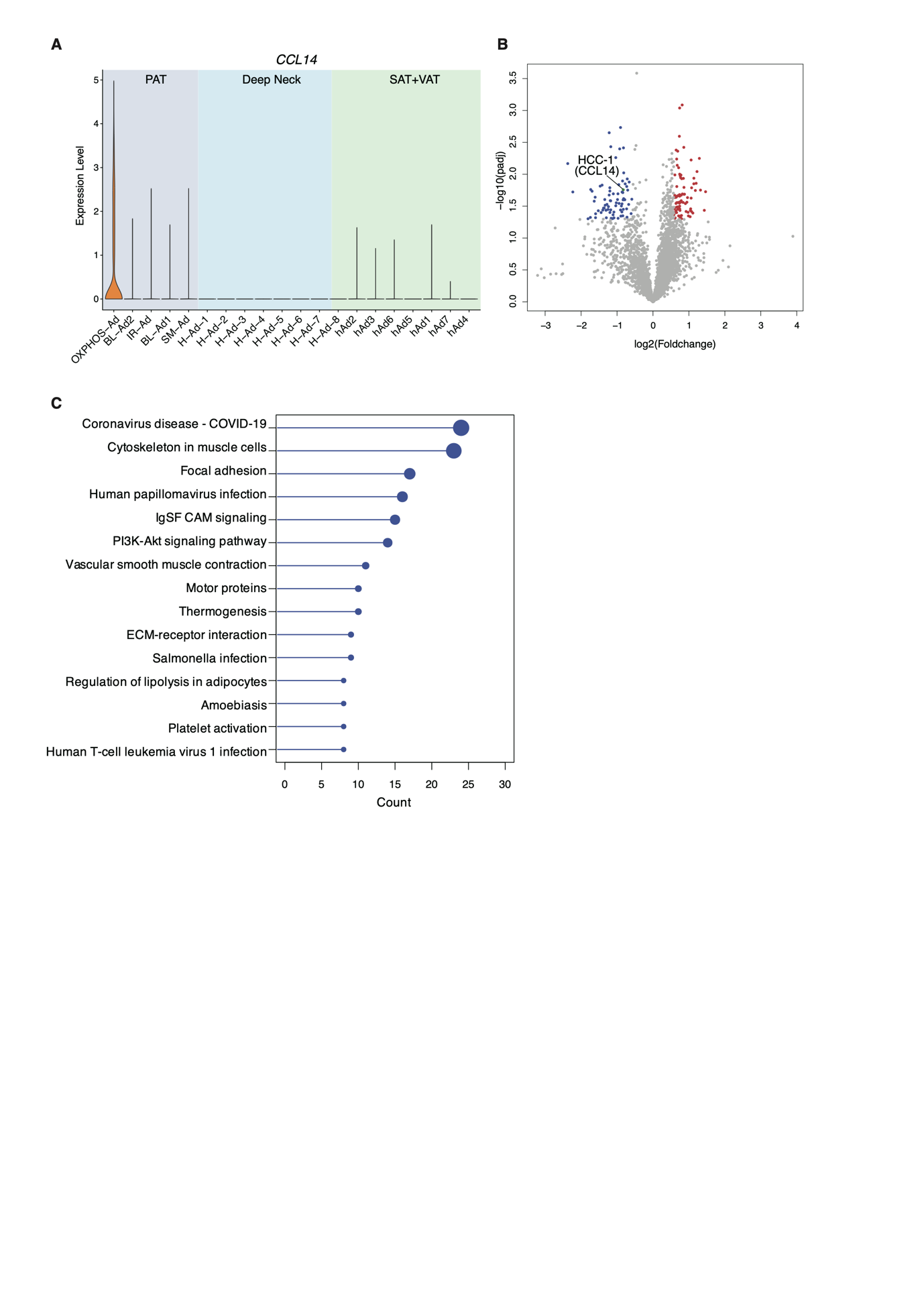
**

**Figure S6. The transcription and protein expression distribution of CCL14 and its receptors CCR1/CCR3/CCR5.** (A)Violin plots showing the expression distribution of CCL14 across different human adipose tissue depots, including PAT, deep neck fat, SAT, and VAT. (B)Volcano plot displaying the differentially abundant secreted proteins in CM collected from PAT explants of patients with PTC compared to those with MNG. (C) Pathway enrichment analysis performed on differentially expressed genes in the OXPHOS-Ad adipocyte subcluster when comparing PTC to MNG patient samples.

**
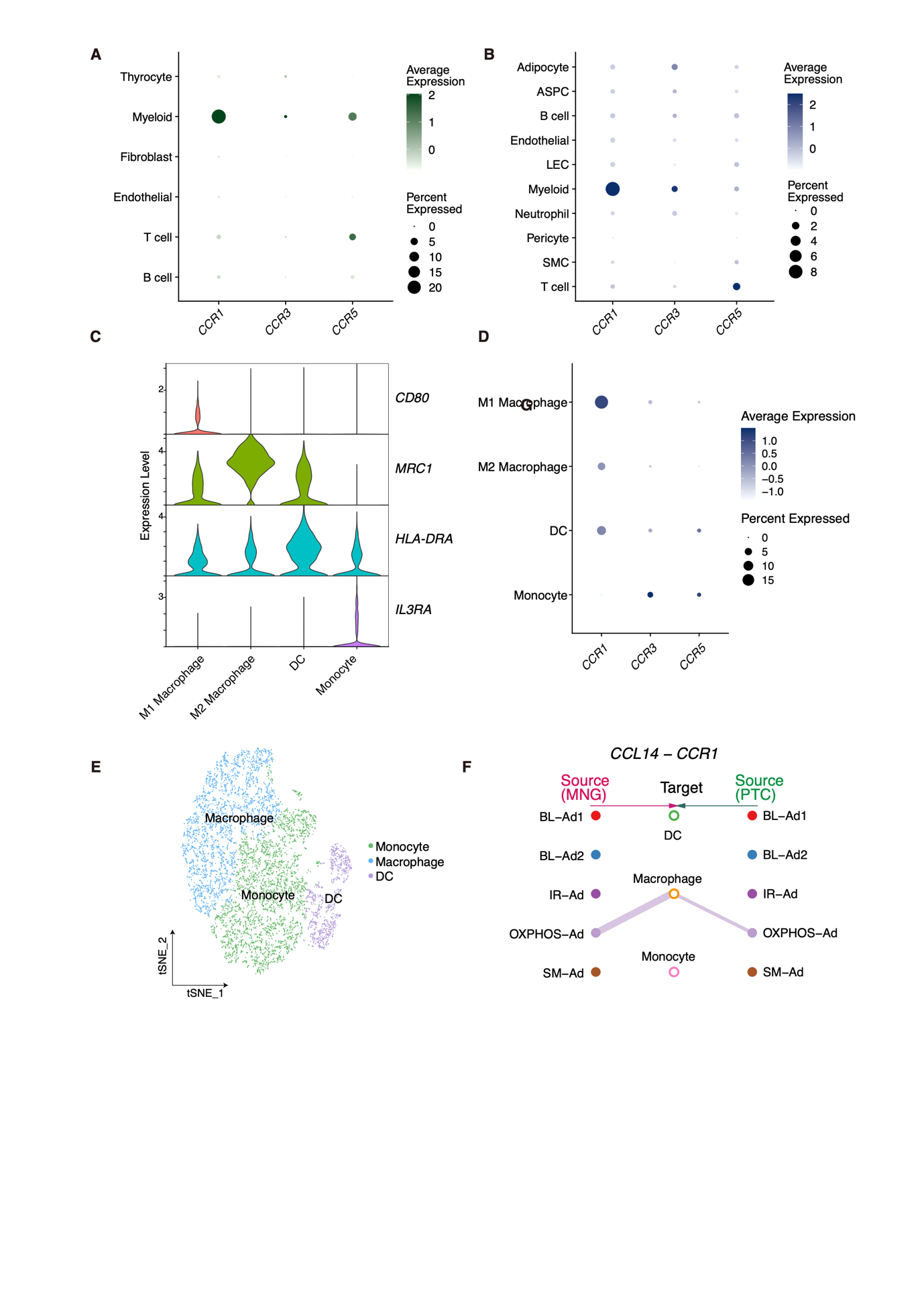
**

**Figure S7. CCR1 is the predominant CCL14 receptor in PAT and thyroid gland.** (A) Dot plot generated from scRNA-seq data of human thyroid tissue, showing the expression levels of known receptors for CCL14 (including CCR1, CCR3, and CCR5) across major thyroid cell types. (B) Dot plot from snRNA-seq data of human PAT, displaying the expression profile of CCL14 receptors across all major cell populations. (C) Violin plot validating the identity of the myeloid cell subclusters in PAT by displaying the expression of canonical marker genes used for their annotation. (D) Dot plot focusing specifically on the expression of CCL14 receptors within the myeloid subpopulations of PAT.(E) t-SNE plot visualizing the subclusters of myeloid cells within the thyroid tissue. (F) Computational prediction of CCL14-CCR1 ligand-receptor interaction strength between adipocyte subclusters in PAT (source of CCL14) and myeloid subclusters within the thyroid gland (expressing CCR1)

**
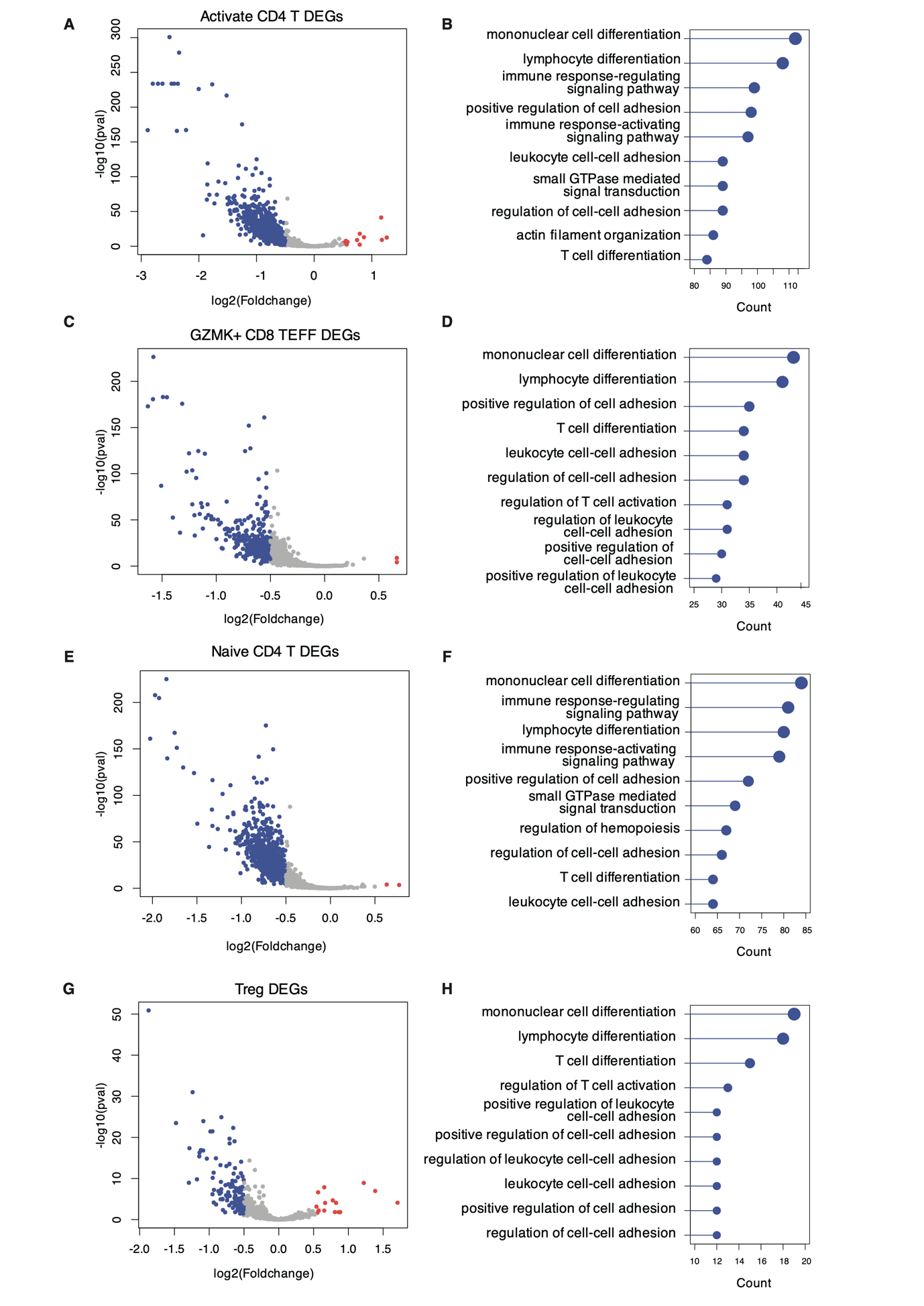
**

**Figure S8. DEGs and GO enrichment of T cell subclusters.** (A) Volcano plot of DEGs in activated CD4 T cell when comparing PAT from PTC patients to MNG controls. (B) Gene Ontology (GO) pathway enrichment analysis performed on the down-regulated DEGs from the Activate CD4 T cell (shown in A).(C) Volcano plot of

| **ID** | **Gender** | **Age** | **BMI** | **Diagnosis** |
| --- | --- | --- | --- | --- |
| **1** | **female** | **41** | **24.0** | **PTC (T1aN0M0)** |
| **2** | **female** | **25** | **23.8** | **PTC (T1aN0M0)** |
| **3** | **female** | **50** | **21.3** | **PTC (T1aN0M0)** |
| **4** | **female** | **25** | **24.6** | **MNG** |
| **5** | **female** | **54** | **23.8** | **MNG** |
| **6** | **female** | **53** | **25.3** | **MNG** |

DEGs in GZMK+ CD8 TEFF cell subcluster when comparing PAT from PTC patients to MNG controls.(D) Gene Ontology (GO) pathway enrichment analysis performed on the down-regulated DEGs from the GZMK+ CD8 TEFF (shown in C).(E) Volcano plot of DEGs in Naïve CD4 T cell subcluster when comparing PAT from PTC patients to MNG controls.(F) Gene Ontology (GO) pathway enrichment analysis performed on the down-regulated DEGs from the Naïve CD4 T cell (shown in E).(G) Volcano plot of DEGs in Treg subcluster when comparing PAT from PTC patients to MNG controls.(H) Gene Ontology (GO) pathway enrichment analysis performed on the down-regulated DEGs from the Treg cell (shown in G).

**Supplementary tables**

Table S1. Patient information

Table S2. Summary of major advancement of the current study

| Aspect | Prior Work | Current Study |
| --- | --- | --- |
| **PAT Identity** | Considered homogeneous and non‑thermogenic | Redefined as immune‑active, thermogenic, and heterogeneous |
| **Cell Lineage** | Developmental trajectories largely unresolved | Distinct ASPCs-to-adipocyte lineages delineated |
| **PAT Function** | ETE as a prognostic marker | PAT triad and CCL14 drive PTC progression, establishing a causal and druggable axis |
